# Cutting and Shuffling Control Mixing in Three-Dimensional Discrete Fracture Networks

**DOI:** 10.1017/jfm.2026.11623

**Published:** 2026-06-10

**Authors:** Stefano Ascione, Daniel R. Lester, Benoit Pinier, Tanguy Le Borgne, Joris Heyman

**Affiliations:** 1 School of Engineering, RMIT University, Melbourne, Australia; 2 Geosciences Rennes, UMR 6118, Université de Rennes 1, CNRS, Rennes, France; 3 Fractory, Itasca consultants, Rennes 35042, France

**Keywords:** Fractured media, advective mixing, discontinuous mixing

## Abstract

Mixing in fracture networks plays a central role in many environmental and geological processes, influencing contaminant dispersion, dilution and biogeochemical reactions. Despite this, fundamental aspects are not well understood, including how the network topology and large fracture aspect ratio governs the stretching, folding, cutting and rearrangement of fluid elements, which represent the advective components of mixing. In this study we focus on three-dimensional (3D) discrete fracture networks (DFNs) governed by steady isotropic Darcy flow. We develop a theoretical framework for advective mixing and uncover two distinct mixing mechanisms at different length scales - fracture mixing and intersection mixing - that arise due to streamline routing in fractures and intersections. We show that the large fracture aspect ratio enforces *discontinuous* mixing, involving cutting and shuffling (CS) of fluid elements due to streamline routing. Mixing is controlled by combined CS and fluctuating fluid deformation, forming a piecewise smooth (PWS) transform and weak ergodic mixing. An efficient graph-based representation of this process is developed via a *mixing graph* ***𝒢_M_*** that efficiently encodes the fracture network topology while capturing mixing dynamics. Numerical predictions of mixing from ***𝒢_M_*** agree to high precision with those from direct numerical simulations. This framework provides new insights into the processes which govern mixing in DFNs and forms a rigorous basis for further application to fluid borne processes such as solute mixing and dispersion, colloidal transport and deposition, chemical reactions and biological activity.

## 1 Introduction

**Figure 1:**
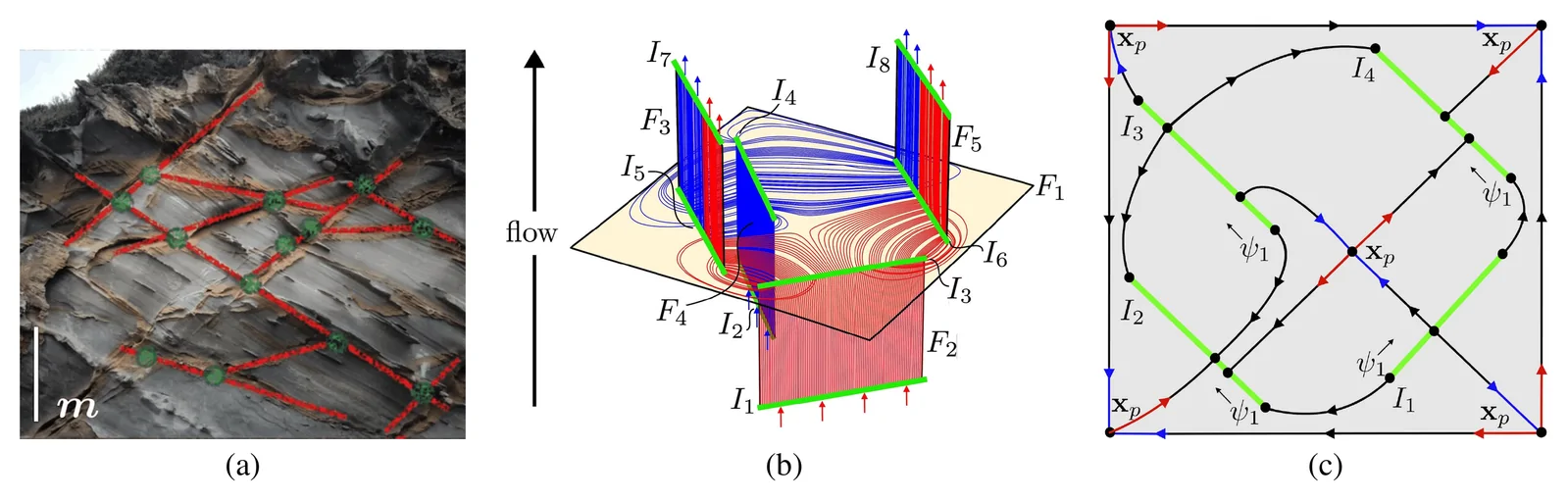
(a) Fractured rock in Cape Otway, Victoria, Australia, with fractures indicated by superimposed red lines and intersections as green dots. Orange bands around fractures indicate regions of chemical activity. (b) Oblique view of a simple DFN comprised of *N*_*F*_ = 5 fractures labelled *f_n_* (*n* = 1 : *N*_*F*_) and *N*_*I*_ = 8 intersections labelled *I*_*m*_, (*m* = 1 : *N*_*I*_ (green lines). Inlet fractures *F*_2_ and *F*_4_ and outlet fractures *F*_3_ and *F*_5_ terminate at and intersect with the central fracture *F*_1_. Streamlines are coloured red or blue respectively depending upon whether they enter via *F*_2_ or *F*_4_ (as indicated by coloured arrows) and are routed through *F*_1_ before exiting via *F*_3_ or *F*_5_ (as indicated by coloured arrows). (c) Plan view of streamline topology in fracture *F*_1_ in (b), indicating routing from “source” intersections *I*_1_, *I*_3_ (green lines) to “sink” intersections *I*_2_, *I*_4_ (green lines) via separating streamlines (black lines) and critical points (black points). Red and blue arrows respectively indicate unstable and stable manifolds. Symbol *ψ*_1_ denotes direction of increasing *ψ*_1_-streamfunction *ψ*_1_ along an intersection.

Solute transport in fractured rock networks plays a central role in a wide range of subsurface applications (Berkowitz 2002), including carbon sequestration (Szulczewski *et al*. 2012), nuclear waste storage (Tsang *et al*. 2015) and geothermal energy extraction (Ren *et al*. 2023). Fractured media, such as that shown in Figure 1a, constitute a particular class of porous material characterised by networks of high-permeability fractures embedded within a comparatively low-permeability rock matrix (Berre *et al*. 2019). These fractures often possess large aspect ratios and arise through a variety of geological processes including tectonic deformation (Bonnet *et al*. 2001), thermo-mechanical stresses (Safari & Ghassemi 2015), and biological activity in the subsurface (Stembridge 1978). Although mixing within fracture systems exerts a first-order control on many fluid-borne processes, including solute dilution (Zhao *et al*. 2014), geochemical reactions (Lee *et al*. 2023), and biological activity (Schuler *et al*. 2024; Bochet *et al*. 2020), flow through fracture networks can generate complex mixing dynamics that are not fully understood (Hyman & Jiménez-Martínez 2018; Hallack *et al*. 2025).

Fracture networks consist of finite-sized three-dimensional (3D) intersecting fractures, often with large aspect ratios. Denoting the characteristic fracture aperture by 𝓁 and the fracture width by *L*, these systems satisfy 𝓁 ≪ *L*. Consequently, flow and transport within individual fractures appear effectively two-dimensional (2D) at the fracture scale *L*, while remaining intrinsically 3D at the aperture scale 𝓁. The narrow geometry of fracture apertures typically renders inertial effects generally negligible, such that the flow is Stokesian at the aperture scale 𝓁 and can often be approximated by Darcy flow at the fracture scale *L*.

**Figure 2:**
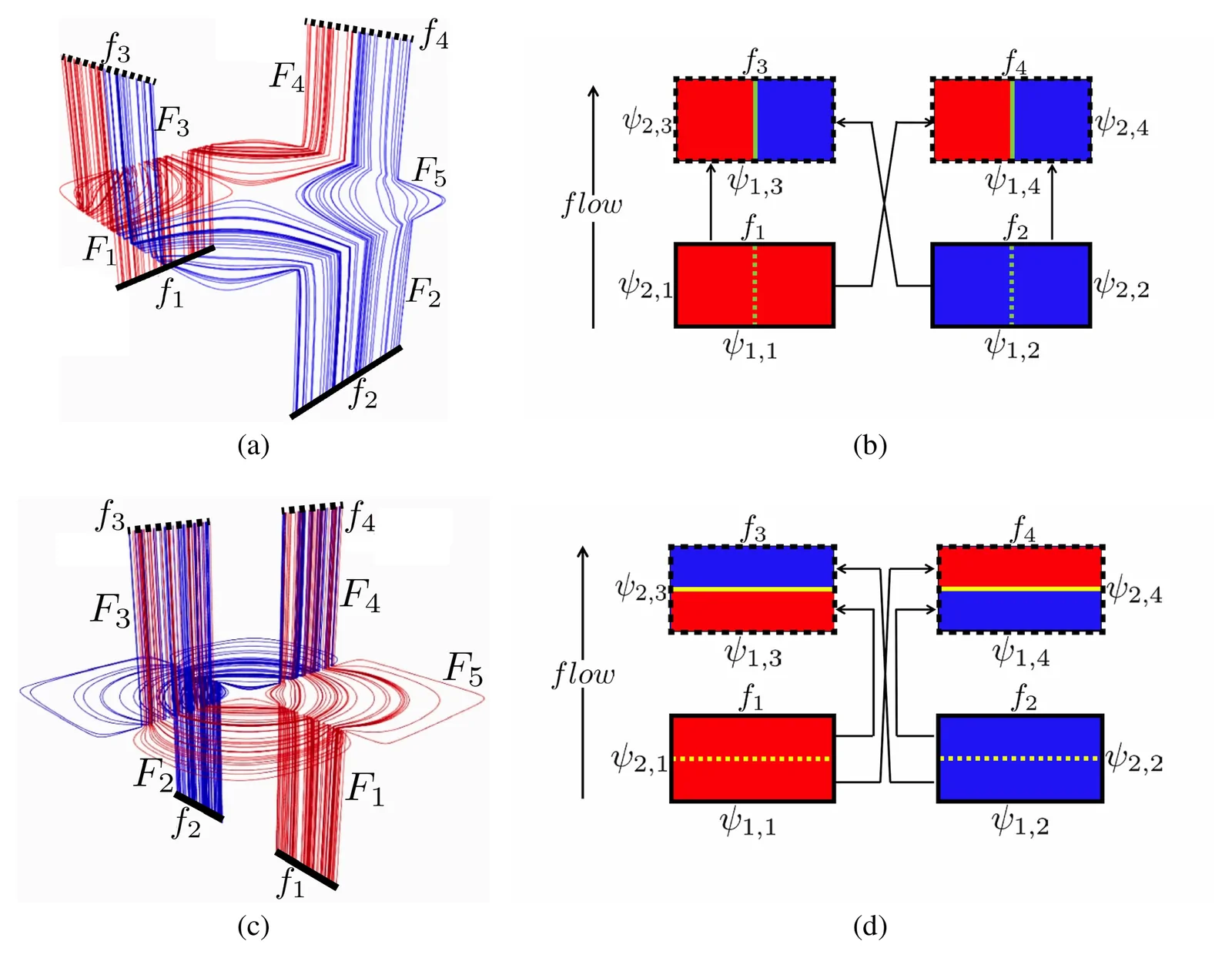
Advective mixing in fracture networks (computed via DFN.lab) comprised of fractures *f_n_* and faces *f_n_* (defined in §§**??** as 2D cross-sections of 3D fractures at their boundary) that exhibit (a,b) fracture mixing only and (c,d) intersection mixing only. (a) Fracture mixing arising from red and blue streamlines entering inlet faces *f*_1_ and *f*_2_. There streamlines are interleaved as “parcels” that exit at outlet faces *f*_3_ and *f*_4_, resulting in large-scale fracture mixing. (b) Mapping of inlet concentration field in faces *f*_1_ and *f*_2_ to *f*_3_ and *f*_4_ via the fracture mixing map ***ℳ_F_*** (3.10) corresponding to the fracture mixing shown in (a) in terms of local streamfunctions (*ψ*_1,*n*_, *ψ*_2,*n*_), *n* = 1 : 4 (defined in §§3.1) at face *f_n_*. (c) Intersection mixing arising from streamline routing at intersections. Red and blue streamlines entering inlet faces *f*_1_ and *f*_2_ are finely interleaved before exiting at outlet faces *f*_3_ and *f*_4_, resulting in fine-scale intersection mixing. (d) Mapping of inlet concentration field in faces *f*_1_ and *f*_2_ to *f*_3_ and *f*_4_ via the intersection mixing map ***ℳ_I_*** (3.9) corresponding to the intersection mixing shown in (c) in terms of local streamfunctions (*ψ*_1,*n*_, *ψ*_2,*n*_) at face *f_n_*.

These geometric considerations motivate the widely used discrete fracture network (DFN) framework, in which fracture systems are represented as a complex of 2D fracture planes connected by 1D intersections embedded within a 3D matrix (Figure 1b) (Jing & Stephansson 2007). In this approach, Darcy flow equations are solved on the fracture surfaces with coupling through the intersections, yielding a computationally tractable representation of flow and transport. Considerable progress has recently been made in understanding solute dispersion within such DFN models (Hyman *et al*. 2015; Hyman 2020; Hyman *et al*. 2022; Dentz & Hyman 2023; Davy *et al*. 2024). However, and outstanding challenge isto understand and predict mixing dynamics within fracture networks (Figure 2).

Although mixing of diffusive species such as solutes and colloids is of direct practical importance, these processes cannot be properly understood without first resolving the underlying advective dynamics (Metcalfe *et al*. 2023). Advective mixing — comprising the stretching, folding, cutting and rearrangement of fluid elements — provides the kinematic foundation upon which diffusion, colloidal transport, chemical reactions and biological processes act (Zhao *et al*. 2014; Lee *et al*. 2023; Schuler *et al*. 2024). Quantitative prediction of these processes therefore requires a detailed understanding of the advective drivers of mixing. † Despite recent progress, the fundamental mechanisms governing advective mixing in fracture networks remain poorly understood.

This gap is reflected in the substantial body of work examining solute mixing at fracture intersections (Berkowitz *et al*. 1994; Park *et al*. 2001; Li 2002; Hyman & Jiménez-Martínez 2018; Sherman *et al*. 2019). Many existing models represent mixing at intersections through flux-weighted random streamline routing, which effectively assumes instantaneous mixing and corresponds to a vanishing Péclet number. Such approaches cannot readily be generalised to finite Péclet numbers and are incompatible with the deterministic and reversible nature of advective transport. Furthermore, because DFN models represent fractures as 2D surfaces connected by one-dimensional (1D) intersections, transverse concentration profiles across the fracture aperture cannot be resolved. These limitations hinder development of analytic models of solute mixing in fracture networks and identification of the network properties that govern mixing.

A central question therefore concerns the extent to which dimensional reduction in DFN models constrains the admissible mixing dynamics. Recent work has begun to quantify mixing metrics such as the growth rate of material interfaces in DFNs (Hallack *et al*. 2025), yet the underlying mechanisms remain unclear. In particular, because the flow within DFN models is restricted to 2D fracture planes, the resulting steady flows must be non-chaotic according to the Poincaré–Bendixson theorem (Katok & Hasselblatt 1995) (despite the 2D fractures being embedded in a 3D matrix). This restriction leads to characteristic non-chaotic mixing signatures, such as the algebraic growth of material interfaces observed in recent studies (Hallack *et al*. 2025). Although it is recognized that topological complexity can generate chaotic advection under steady flow in pore networks and granular matter Lester *et al*. (2025*a*), it is unclear to what extent these dynamics are hindered by the constrained geometry inherent to fracture networks.

While parsimonious representations of transport, mixing and reactions in fracture networks are essential for large-scale modelling, properly resolving these 3D mixing mechanisms is equally important. Representing fractures as strictly 2D objects constrains the admissible Lagrangian kinematics, suppresses the influence of fracture roughness on transport and mixing, and may eliminate key processes such as fluid stretching and chaotic advection. Once these mixing mechanisms are resolved, it is then possible to develop faithful reduced-order representations of fluid borne-process in fracture networks.

The aim of this study is therefore to elucidate and quantify the advective dynamics that govern mixing in 3D DFNs in which fractures possess finite thickness. We consider the propensity for complex advective mixing dynamics such as chaotic advection and discontinuous mixing to arise in these networks, and develop an appropriate Lagrangian framework to represent advective mixing. Building on this understanding, we develop a highly accurate yet computationally efficient framework for modelling advective mixing in in 3D DFNs which is verified against numerical simulations performed using the open source code DFN.lab (Le Goc *et al*. 2019), https://fractorylab.org/dfnlab-software. Although DFN.lab is a 2D DFN code, we show that via the use of a streamfunction coordinate system (the validity of which is established in §§ 2.3), that the 3D flow field (in streamfunction coordinates) can be reconstructed from these 2D simulations via local flux balances (see Appendix B). This proposed approach provides a basis for identification of the network properties that govern advective mixing, development of quantitative mixing theories for large-scale networks and facilitate prediction of advection-mediated processes including solute dispersion, mixing and biogeochemical reactions in 3D fracture networks. Similar to 2D DFN models, the flow in 3D DFNs is assumed to be steady and governed by isotropic Darcy dynamics throughout the fracture network. The implications of these assumptions are examined in §§2.1. The paper is structured as follows. In §2, we develop a streamline coordinate system for advective mixing which is used to uncover the fundamental mixing mechanisms in 3D DFNs. In §3 we use the streamline coordinate system to construct a parsimonious representation of advective mixing in 3D DFNs, and in §4 we verify this model against fully resolved DFN simulations. Conclusions and implications are discussed in §5.

## 2 Advective Mixing in Fracture Networks

### 2.1 Chaotic Advection in Fracture Networks

To analyse advective mixing in fracture networks, we first consider the propensity for these systems to admit complex mixing dynamics, and use this assessment to develop a parsimonious representation of advective mixing. We focus on fracture networks at negligible Reynolds number, *Re* ≪ 1, with large fracture aspect ratio 𝓁 ≪ *L*. Under these conditions inertia-driven mixing mechanisms, such as those that arise at fracture intersections (Lee & Kang 2020; Yang *et al*. 2024), are absent. However, complex mixing dynamics may also arise in inertialess flows in topologically complex domains.

**Figure 3:**
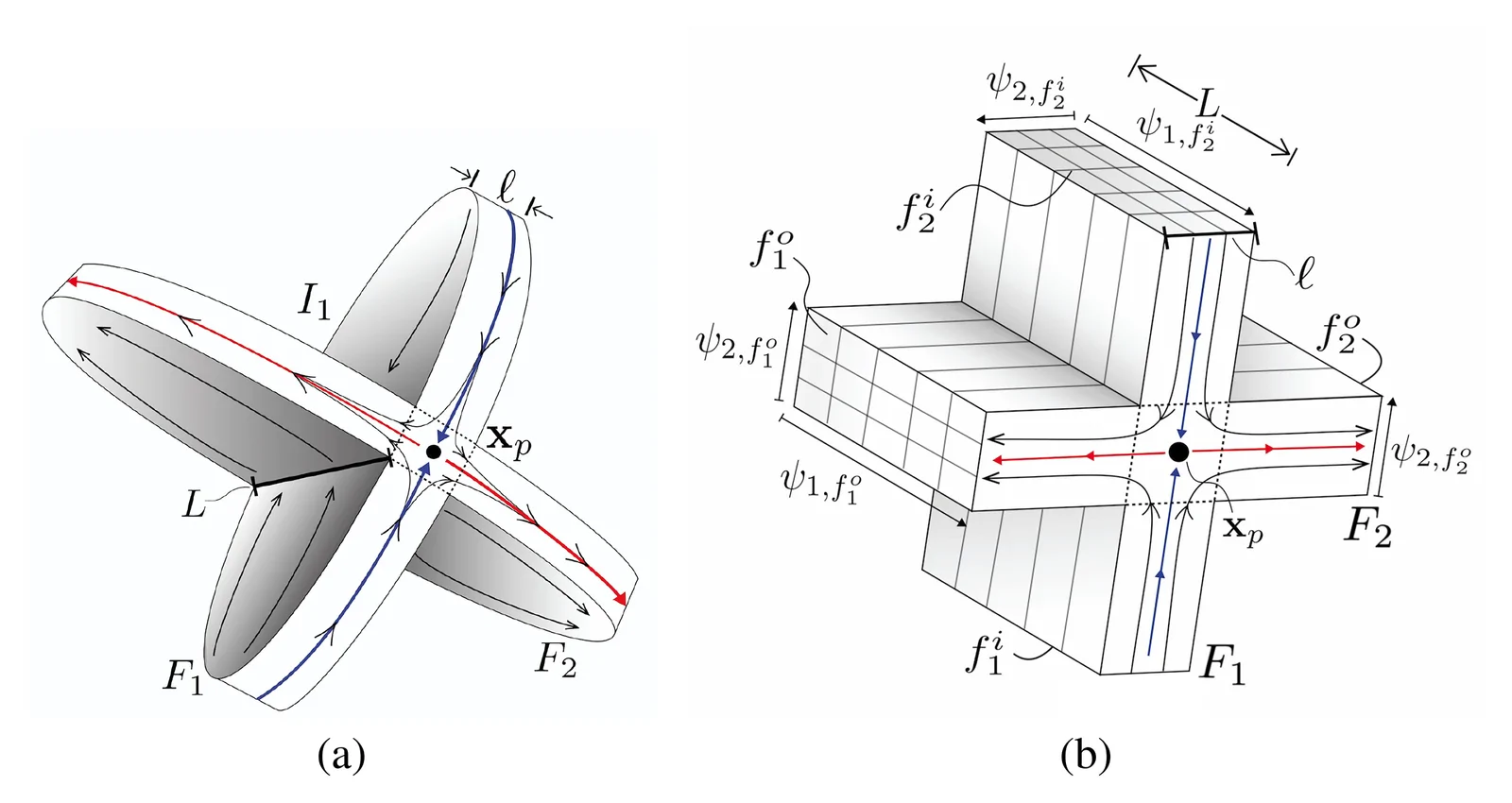
(a) Schematic of two intersecting fractures *F*_1_, *F*_2_ of characteristic aperture 𝓁 and width *L* forming an intersection *I*_1_. Streamlines are separated by a continuous line of hyperbolic stagnation points **x**_*p*_ at the fracture intersection, and the associated stable (blue) and unstable (red) hyperbolic manifolds route surrounding streamlines (black) to their respective outlet faces. (b) Schematic detailing intersection *I*_1_ shown in (a) depicting the inlet faces $f_{1}^{i},f_{2}^{i}$ associated with fracture *F*_1_ and outlet faces $f_{1}^{o},f_{2}^{o}$ associated with fracture *F*_2_. Also shown are the local streamfunctions *ψ*_1,*n*_ ∈ [0, 1], *ψ*_2,*n*_ ∈ [0, 1] and associated *ψ*_1,*n*_- and *ψ*_2,*n*_-streamsurfaces that are respectively oriented along the fracture width *L* and fracture aperture 𝓁 of each face. Similar to (a), the hyperbolic point **x**_*p*_ and associated manifolds route streamlines into downstream outlet faces. Local streamfunctions *ψ*_2_ govern routing of streamlines, which leads to cutting and shuffling of fluid elements leaving $f_{1}^{i},f_{2}^{i}$ and arriving at $f_{1}^{o},f_{2}^{o}$.

**Figure 4:**
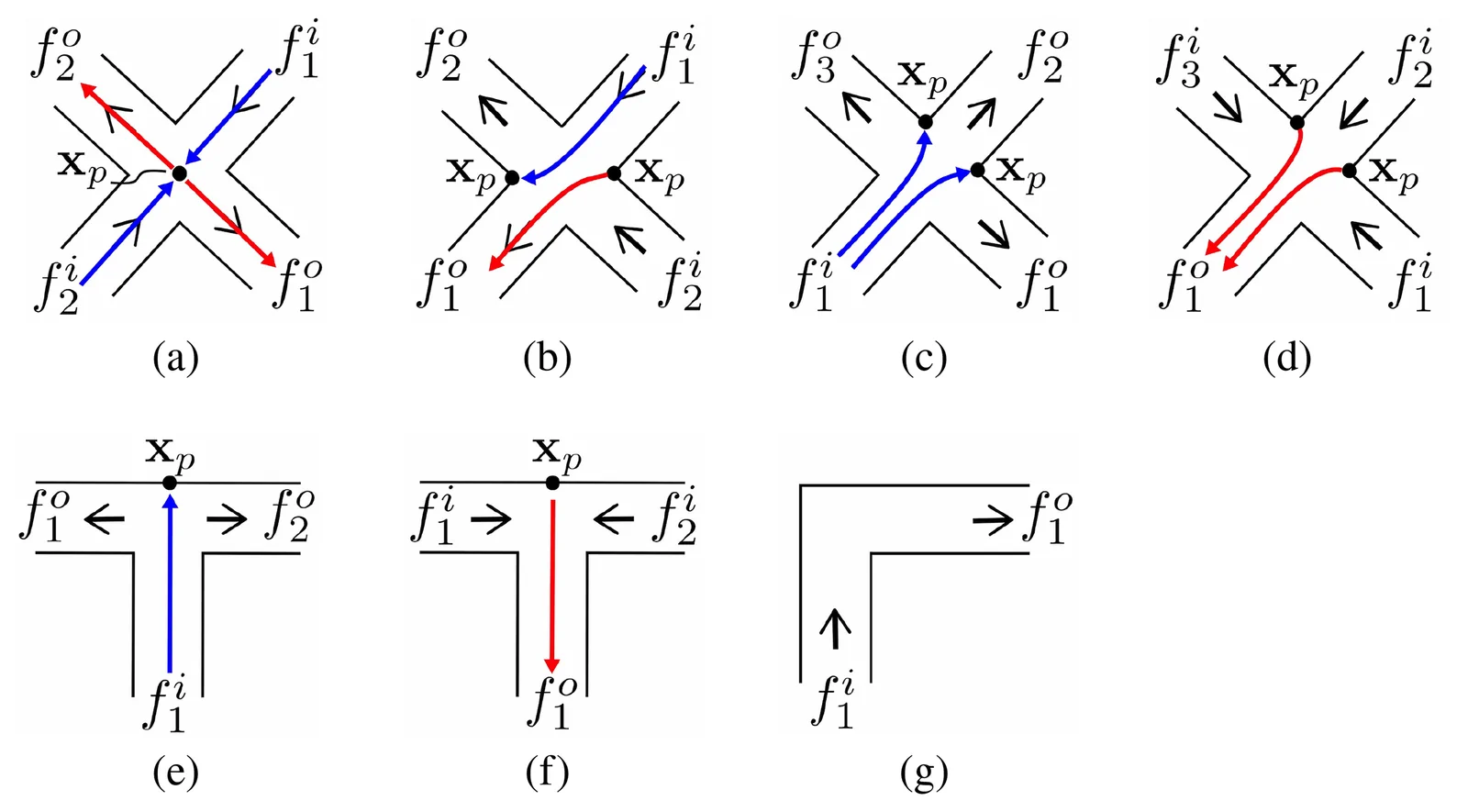
Schematic of the complete set of distinct streamline topologies in a cross-section (a-d) *X*-, (e-f) *T*- and (g) *L*-shaped intersections. Each cross-section corresponds to a ${\hat{\psi }}_{1}$ streamsurface, hence the streamline topology is the same as that for a steady 2D flow. Stagnation points **x**_*p*_ and corresponding (blue) stable and (red) unstable hyperbolic manifolds (which act as separating streamlines) act to route fluid parcels through inlet $f_{n}^{i}$ and outlet $f_{n}^{o}$ faces of the intersection. Black arrows indicate flow direction.

**Figure 5:**
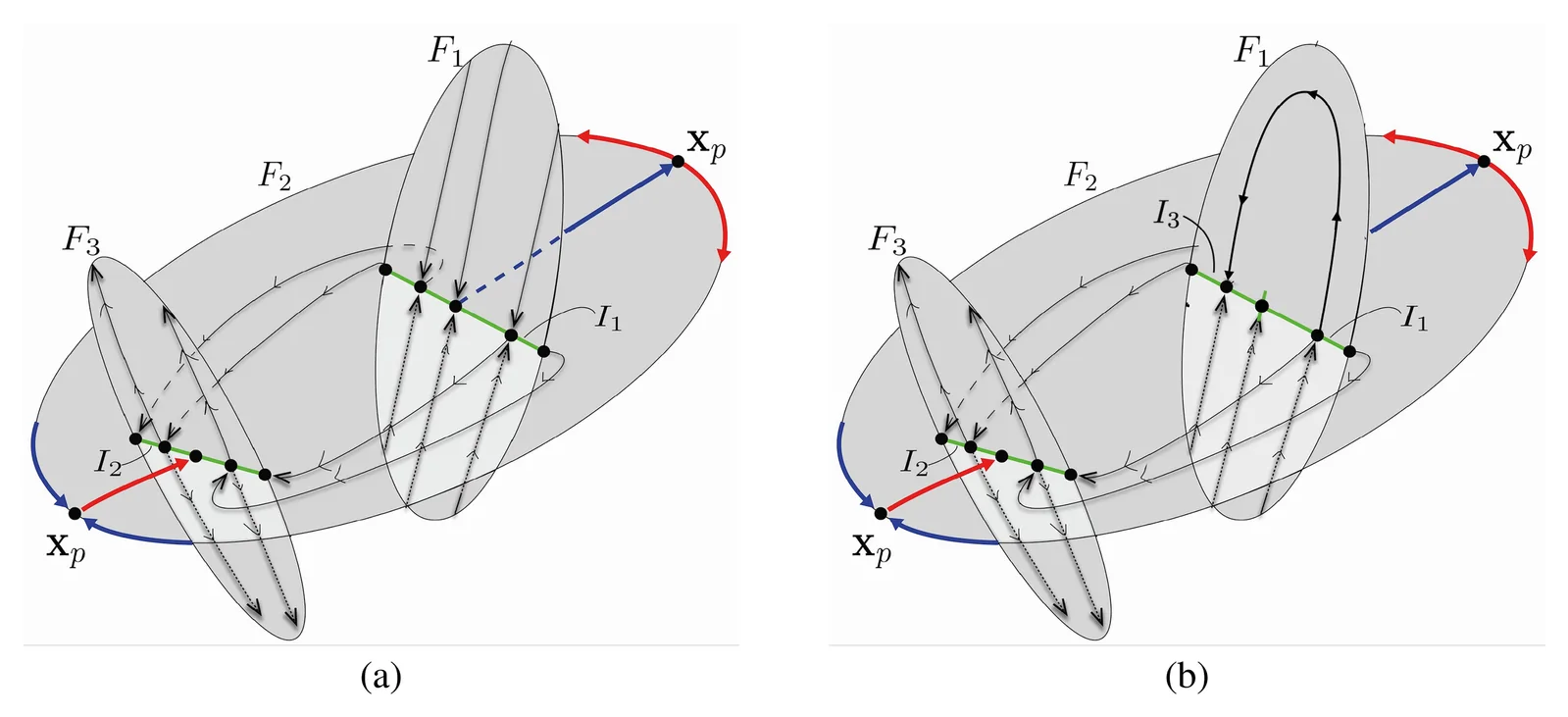
(a) Schematic of a simple fracture network (at the fracture scale *L*) comprised of fractures *f_n_* (where *F*_1_ and *F*_3_ respectively are inlet and outlet fractures) and intersections *I*_*m*_ (green lines), separating streamlines (black lines), critical points (black dots). Separating streamlines route fluid “parcels” from “source” intersection faces to different “sink” intersection faces. Stagnation (critical) points are denoted **x**_*p*_ (with corresponding hyperbolic stable (blue) and unstable (red) manifolds), while terminal and interior critical points are unlabelled. All separating streamlines either originate or terminate at stagnation or terminal critical points. (b) Same schematic as (a) but with a dead-end fracture (*F*_1_) which is treated by splitting intersection *I*_1_ in (a) into an “outflow” intersection *I*_1_ and an “inflow” intersection *I*_3_, with streamlines routed in *F*_1_ from *I*_1_ to *I*_3_.

In the Stokes regime, steady flow in fracture networks may generate chaotic advection in a manner analogous to porous networks or granular media (Lester *et al*. 2025*a*, 2013). Pore and fracture networks both involve merging and branching of streamlines at pore junctions and fracture intersections respectively, giving rise to localised stagnation points **x**_*p*_ and associated unstable and stable 2D hyperbolic manifolds; material surfaces that respectively undergo exponential stretching and contraction which are shown in Figures 1c, 3, 4, 5 and 7. If these manifolds intersect transversely, fluid elements undergo exponential stretching and folding, leading to chaotic advection (Ottino 1989). Indeed, several studies (Mourzenko *et al*. 2002; Johnson *et al*. 2006; Zhao *et al*. 2014) have examined steady 3D Stokes flow in wide and rough fracture intersections and found that although chaotic advection was not explicitly diagnosed, velocity fields were observed that exhibited 3D structure consistent with non-trivial streamline braiding and thus chaotic advection (Boyland *et al*. 2000). Conversely, tangential intersections between 2D manifolds suppress chaotic advection as the exponential expansion and contraction cancel. As fracture networks comprised of narrow fractures impose strongly constrained geometries that favour near-tangential manifold intersections, the prevalence of chaotic advection under steady Stokes flow is unclear.

The common assumption of isotropic Darcy flow in 3D DFNs further constrains the Lagrangian kinematics. Within fractures, steady 3D Stokes flow may be validly approximated as a Hele-Shaw flow with variable aperture *h*(**x**) ~ 𝓁 across the fracture. Hence for 𝓁 ≪ *L*, the flow in fractures can be validly approximated as an isotropic heterogeneous Darcy flow (Bear 1972)

(2.1) $$\text{v}(\text{x})=-\frac{k(\text{x})}{\mu }\nabla p(\text{x}),$$

where *µ* is the fluid viscosity and the permeability *k*(**x**) = *h*(**x**)^2^/12. For 2D and 3D DFNs, it is assumed that isotropic Darcy flow extends throughout the entire fracture network. This renders the velocity field *helicity-free* (Sposito 2001; Lester *et al*. 2021), where from (2.1) the helicity density (Moffatt 1969), defined as ℋ (**x**) ≡ **v** · (**∇** × **v**), vanishes identically as

(2.2) $$\text{ℋ}(\text{x})\equiv \text{v}(\text{x})\cdot (\nabla \times \text{v}(\text{x}))=\frac{1}{\mu^{2}}k(\text{x})\nabla p(\text{x})\cdot (\nabla k(\text{x})\times \nabla p(\text{x}))=0.$$

Although anisotropic 3D Darcy flow relaxes this constraint (Lester *et al*. 2025*b*), such formulations have not, to our knowledge, been implemented in fracture network models. For isotropic Darcy flow the condition ℋ(**x)** = 0 implies that the flow is *globally integrable* (i.e. non-chaotic) in simply connected domains in the sense of Arnol’d (1965). However, this integrability may not persist in multiply connected domains such as fracture networks.

Beyond fracture networks, the question of whether chaotic advection can arise under steady isotropic 3D Darcy flow in topologically complex (multiply-connected) domains is relevant to many applications, including transport processes in groundwater systems, resin transfer moulding and packed-bed reactors. Although Camacho & Neto (1981) showed that closed (recirculating) streamlines in multiply connected domains can break global integrability, such streamlines cannot arise in Darcy flow (Bear 1972). Nevertheless, multiply connected domains typically contain numerous stagnation points according to the Poincaré–Hopf theorem (Poincaré 1881, 1882; Hopf 1927). At such points the Frobenius theorem (Frobenius 1877) – which links zero helicity density to integrability – breaks down, meaning the flow is only *locally* integrable. Consequently, both steady 3D Stokes flow and isotropic Darcy flow in multiply connected domains may admit chaotic advection through fluid deformation at stagnation points. However, the zero helicity density nature of isotropic Darcy flow means that streamline braiding (another potential mechanism for chaotic advection) can only occur in Stokes flow.

The degree of chaotic advection in fracture networks due to stagnation points may be estimated using a simple model for the dimensionless Lyapunov exponent $\lambda_{\infty }\equiv {\hat{\lambda }}_{\infty }\tau_{v}$ developed for random porous networks (Lester *et al*. 2013), where ${\hat{\lambda }}_{\infty }$ is the dimensional Lyapunov exponent and *τ_v_* is the mean advection time between fracture intersections. In Appendix A.1 we apply this model to fracture networks with aspect ratio 𝓁/*L* and obtain the estimate λ_∞_ ≈ 𝓁/(8*L*) ≪ 1, which is substantially smaller than those reported for random porous networks (λ_∞_ ≈ 0.1178) or granular media (λ_∞_ ≈ 0.17–0.21) (Heyman *et al*. 2020; Souzy *et al*. 2020; Heyman *et al*. 2021). Similarly, streamline braiding under Stokes flow conditions is also expected to be weak for 𝓁 ≪ *L* as helicity is shown in Appendix A.2 to be localised to boundaries. This suggests that the strongly constrained geometry of fracture networks renders chaotic advection negligible, although further investigation is required to establish this conclusion more firmly.

### 2.2 Model Scope and Assumptions

Accordingly, we assume chaotic advection is negligible in large aspect ratio (𝓁 ≪ *L*) fracture networks. For simplicity of exposition we also assume that intersections involve at most two fractures (precluding higher-order junctions) and that pressure gradients along intersections are negligible. To account for dead-end fractures which arise from pressure gradients along intersections (and play an important role in advective transport (Kang *et al*. 2020; Yoon *et al*. 2023)), we partition each intersection into “inflow” and “outflow” segments relative to the dead-end fracture. This yields a piecewise-constant approximation of the pressure gradient along intersections (§§ 2.4).

Under these assumptions, advective mixing is both deterministic and reversible and is governed entirely by streamline organisation within the network. To quantify this process, we consider both streamline routing (§§ 2.3, 2.4) and the steady transport of a continuously injected, non-diffusive tracer. As tracer concentration is conserved along streamlines, mixing is controlled solely by their transverse organisation, and is independent of the Lagrangian travel time *τ*. Consequently, shear-driven mechanisms (e.g. Taylor–Aris dispersion) and purely advective longitudinal dispersion (Dentz & Hyman 2023) are neglected. The present framework can, however, be extended (through charac-terisation of the Lagrangian travel time statistics (Davy *et al*. 2024)) to incorporate time-dependent processes such as solute diffusion and reaction (§ 5).

Resolving streamline dynamics at both the aperture scale 𝓁 and fracture scale *L*, we show that advective mixing is governed by streamline routing through fractures and their intersections (§§ **??**), leading to *discontinuous* mixing via cutting-and-shuffling (CS) of fluid elements (Sturman 2012; Park *et al*. 2016; Smith *et al*. 2017), rather than the stretching-and-folding (SF) mechanisms characteristic of chaotic advection in porous media (§§ 2.6). We also assess the contribution of fluctuating fluid deformation superimposed on this process.

This discontinuous mixing is naturally represented by a graph-based formulation (§ 3), the *mixing graph* ***𝒢_M_***, which extends the standard DFN intersection graph ***𝒢_I_*** (Hyman *et al*. 2017). Comparison with fully resolved DFN simulations in § 4 demonstrates that ***𝒢_M_*** accurately captures advective mixing, supporting both the discontinuous mixing mechanism and its parsimonious graph representation. By linking mixing dynamics directly to network topology, ***𝒢_M_*** enables quantitative analysis of advective mixing in large random networks using both numerical and analytical approaches. This also provides a foundation for *ab initio* modelling of time-dependent transport processes, including diffusion, reactions and colloidal transport.

### 2.3 Streamfunction Coordinate System

To resolve advective mixing in 3D DFNs as a deterministic and reversible process, we quantify streamline routing via a streamline coordinate system, the existence of which is guaranteed if ℋ (**x**) = 0, which ensures the existence of a *holonomic* (coherent) (Schutz 1980) coordinate frame. As the 3D DFN velocity field **v**(**x**) is globally integrable, it may be expressed in terms of the dual streamfunctions (Lester *et al*. 2023)

(2.3) $$\text{v}(\text{x})=Q\nabla {\hat{\psi }}_{1}(\text{x})\times \nabla {\hat{\psi }}_{2}(\text{x}),$$

where *Q* is the total volumetric flow rate through the fracture network and the streamfunctions $\left({\hat{\psi }}_{1},{\hat{\psi }}_{2}\right)$ are orthogonal $\left(\nabla {\hat{\psi }}_{1}\cdot \nabla {\hat{\psi }}_{2}=0\right)$ and normalised $\left({\hat{\psi }}_{1}\in [0,1],{\hat{\psi }}_{2}\in [0,1]\right)$. As the dual streamfunctions are non-unique (Lester *et al*. 2021), we define without loss of generality that the ${\hat{\psi }}_{1}$ and ${\hat{\psi }}_{2}$ streamfunctions respectively vary along the fracture width (~ *L*) and aperture (~ 𝓁).

The dual streamfunctions are analogous to the single streamfunction *ψ(x*_1_, *x*_2_) for a 2D incompressible flow, $\text{v}=\nabla \psi \times {\hat{\text{e}}}_{3}$, which may be extruded into a 3D flow in the *x*_3_ direction as **v** = **∇***ψ*_1_× **∇***ψ*_2_, with *ψ*_1_ = *ψ* and *ψ*_2_ = *x*_3_. Hence the dual streamfunction representation provides a natural 3D extension of 2D incompressible flow (Bear 1972). In both cases the flow is globally integrable as there exist *d*-1 (where *d* is Eulerian dimension of the flow) *invariants* of the flow given by the streamfunctions (as $\text{v}\cdot \nabla {\hat{\psi }}_{1}=\text{v}\cdot \nabla {\hat{\psi }}_{2}=0$).

As the streamfunctions ${\hat{\psi }}_{1},{\hat{\psi }}_{2}$ are invariant, streamlines are confined to coherent 2D streamsurfaces ${\hat{\psi }}_{1}$ = const., ${\hat{\psi }}_{2}$ = const. (henceforth termed ${\hat{\psi }}_{1}$ - and ${\hat{\psi }}_{2}$-streamsurfaces), and the flow is termed *epi*-2D (Yoshida & Morrison 2017). Hence 1D streamlines arise at the intersection of ${\hat{\psi }}_{1}$– and ${\hat{\psi }}_{2}$–streamsurfaces, and so each 1D streamline may be uniquely labelled as the streamfunction pair $\left({\hat{\psi }}_{1},{\hat{\psi }}_{2}\right)$.

From (2.1) and (2.3) the fluid pressure gradient **∇***p* (**x**) is also orthogonal to the streamfunctions $\left(\nabla p\cdot \nabla {\hat{\psi }}_{i}=0,i=1,2\right)$, and so provides a structured, orthogonal 3D Lagrangian coordinate system $\text{ζ}\equiv \left(\zeta_{1},\zeta_{2},\zeta_{3}\right)\equiv \left({\hat{\psi }}_{1},{\hat{\psi }}_{2},p\right)$. The transform from Eulerian space **x** = (*x*_1_, *x*_2_, *x*_3_) to ***ζ*** completely quantifies advective mixing. We note that the coordinate system ***ζ*** naturally resolves advective mixing in rough fractures and intersections of variable aperture fractures as the 2D boundaries of 3D fractures correspond to coherent ${\hat{\psi }}_{1}$- or ${\hat{\psi }}_{2}$-streamsurfaces. Streamline coordinates will be utilized extensively to quantify advective mixing in 3D DFNs.

### 2.4 Intersection and Fracture Mixing

We consider simple 3D DFNs (Figure 2) as a basis for methods that extend to large networks. Despite their simplicity, these systems exhibit two distinct mixing mechanisms controlled by fracture geometry and connectivity. In the first configuration, streamline routing within fractures produces large-scale mixing at the fracture length scale *L*, whereas in the second, routing at intersections induces fine-scale mixing at the aperture scale 𝓁. These define *fracture mixing* (at length scale *L*) and *intersection mixing* (at length scale 𝓁).

We denote the 2D cross-section immediately adjacent to an intersection as a face *f_n_* (*n* = 1:*N*_*f*_). Under the assumption negligible longitudinal pressure gradients along intersections, the velocity field **v**(**x**) is orthogonal to all faces *f_n_*. Hence each face is classified as an inlet $f_{n}^{i}$ or outlet $f_{n}^{o}$ relative to its adjacent intersection; by convention, we define an inflow face (i.e. that with fluid flowing into the network) to be an outlet face $f_{n}^{o}$, and likewise an outflow face (i.e. that with fluid flowing out of the network) to be an inlet face $f_{n}^{i}\;$. Streamlines thus traverse the network periodically, encountering outlet faces, fractures, inlet faces, and intersections in sequence.

As shown in Figure 3, intersection mixing is governed by streamline routing between inlet and outlet faces. Owing to the epi-2D nature of isotropic Darcy flow, streamlines lie on ${\hat{\psi }}_{1}$-streamsurfaces and exhibit the topology of steady 2D planar flow. Within each surface, routing is determined by separatrices connected to the stagnation point **x**_*p*_. This topology is invariant across ${\hat{\psi }}_{1}$-streamsurfaces, such that 0D stagnation points and 1D separatrices correspond to 1D stagnation lines and 2D separating ${\hat{\psi }}_{1}$-streamsurfaces spanning the 3D intersection. Intersections of two fractures may also form *X*-, *T*-, or *L*-shaped cross-sections, with four, three, or two faces, respectively (Figure 4). These admit seven distinct planar streamline topologies, distinguished by inlet–outlet configuration and stagnation structure. As only ${\hat{\psi }}_{2}$ varies within each ${\hat{\psi }}_{1}$-streamsurface, it fully characterises streamline routing during intersection mixing.

Similar to intersection mixing, fracture mixing is driven by the routing of streamlines within fractures to different faces *f_n_*. As streamlines are confined to ${\hat{\psi }}_{2}$-streamsurfaces (Figure 5a), streamline topology within each fracture is the same as that of a 2D planar flow. Within each ${\hat{\psi }}_{2}$-streamsurface, streamlines are routed by separating streamlines connected to stagnation points and other critical points (defined in §§ 2.5). Similar to intersection mixing, these 0D critical points and 1D separating streamlines manifest as 1D critical lines and 2D separating ${\hat{\psi }}_{1}$-streamsurfaces across the full 3D fracture.

**Figure 6:**
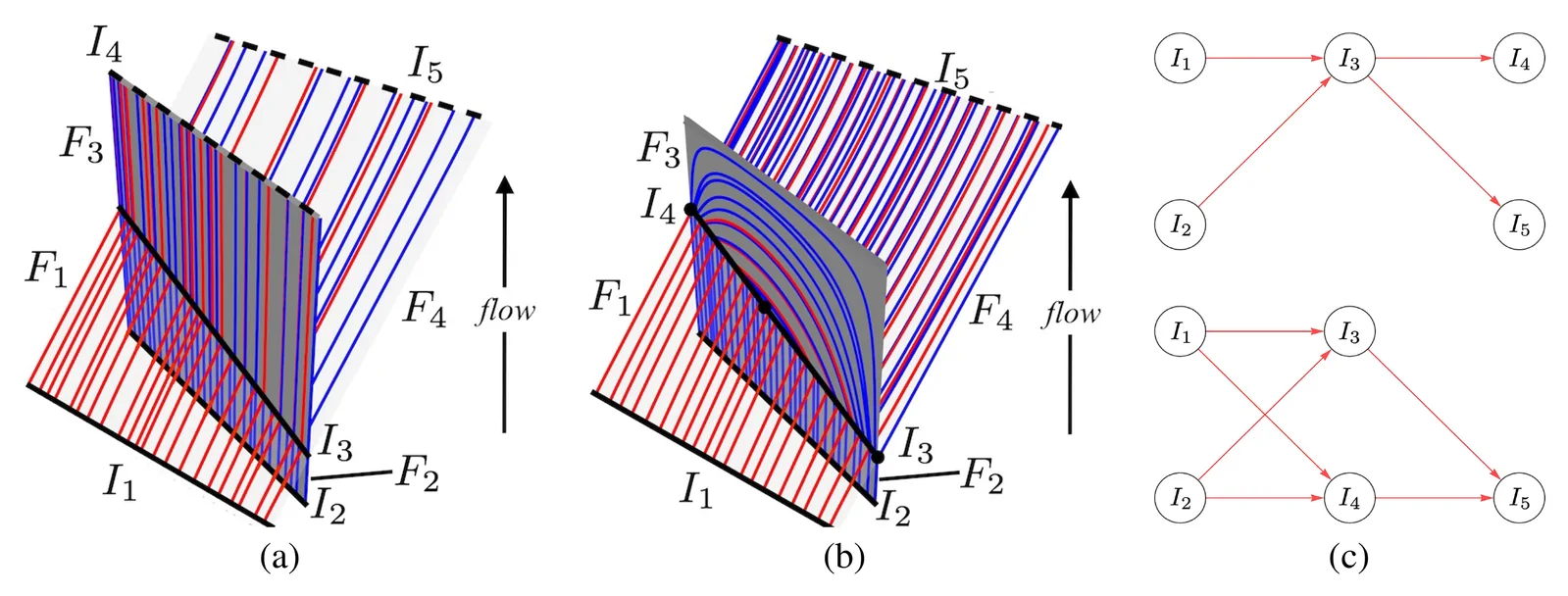
(a) Random, flux-weighted routing of streamlines through an *X*-shaped intersection, computed using DFN.lab. Note that random routing directs both red and blue inlet streamlines into both outlet fractures, which cannot occur for deterministic streamlines. (b) Random, flux-weighted routing of streamlines through a dead-end fracture *F*_3_, computed using DFN.lab. Note that intersection *I*_3_ in (a) can be split into two separate fractures *I*_3_, *I*_4_. (c) Intersection graphs ***𝒢_I_*** depicting (top) the *X*-shaped intersection shown in (a) and (bottom) the *X*-shaped intersection with dead-end shown in (b).

As shown in Figure 5a, even simple fractures (e.g. *F*_2_) connecting a single source (*I*_1_) and sink (*I*_2_) contain multiple separatrices that partition flow between faces, yielding generally more complex topologies than for intersection mixing (Figure 4). Figure 5b illustrates the treatment of dead-end fractures under the assumption of constant pressure along intersections. By splitting the intersection *I*_1_ in Figure 5a into “inflow” and “outflow” components (with respect to the dead-end fracture *F*_1_), streamlines are routed from *I*_1_ to *I*_3_ via the same mechanism as in non-dead-end fractures. Figure 6a shows typical streamlines (computed using DFN.lab) for an *X*-shaped intersection. The flux-weighted random routing in DFN.lab distributes red and blue streamlines across both outlets, whereas deterministic routing (Figure 4b) directs all streamlines of a given type into at least one outlet. This discrepancy highlights the limitations of random routing for representing advective—and thus finite-*Pe*—mixing. Figure 6b shows the corresponding routing in a dead-end fracture, where *I*_3_ is split into *I*_3_ and *I*_4_; the resulting intersection graphs are shown in Figure 6c, with intersections as nodes and fractures as edges.

Despite the differences in complexity of streamline topology and the presence of dead-end fractures, fracture mixing is very similar to intersection mixing in that it is driven by streamline routing. Hence critical points, lines and surfaces control both intersection and fracture mixing in 3D DFNs.

### 2.5 Critical Points, Lines and Surfaces

The inherent topological complexity of fracture networks gives rise to critical features - stagnation points and lines, separating streamlines and streamsurfaces (Figures 3, 4, 5) - that control streamline routing and advective mixing. In 3D DFNs, stagnation points **x**_*p*_ arise at junctions within fractures, which are connected to separating streamlines that route fluid parcels to/from different intersections within the fracture. In addition to stagnation points, terminal critical points shown (Figure 5a) arise at the termini of 1D intersections and differentiate flows into or out of different faces *f_n_* on each side of an intersection. Hence, in fracture flows there are additional separating streamlines that originate or terminate at terminal critical points, and these separating streamlines can either originate or terminate at stagnation points, terminal points or midway along faces *f_n_*, forming the interior critical points shown in Figure 5a. Together, these critical points and their associated separating streamlines act to route fluid elements from parts of outlet faces $f_{n}^{o}$ to parts of inlet faces $f_{n}^{i}$, leading to discontinuous mixing as discussed in the following.

### 2.6 Discontinuous Mixing in Fractured Media

Although stretching and folding (SF) of fluid elements around stagnation points in 3D DFNs cannot generate significant chaotic advection, it does generate *discontinuous mixing* (Smith *et al*. 2019; Sturman 2012), involving cutting-and-shuffling (CS) of fluid elements. CS is akin to shuffling of a deck of infinitely divisible cards, where an initially continuous set of streamlines is “cut” into a set of streamline “parcels” that are then “shuffled” and recombined in different arrangement. In the language of ergodic theory (Arnold & Avez 1968), mixing via CS is *weak mixing*, as compared to *strong mixing* under SF, however CS mixing can still lead to rapid and complete (ergodic) advective mixing (Sturman 2012; Smith *et al*. 2019).

**Figure 7:**
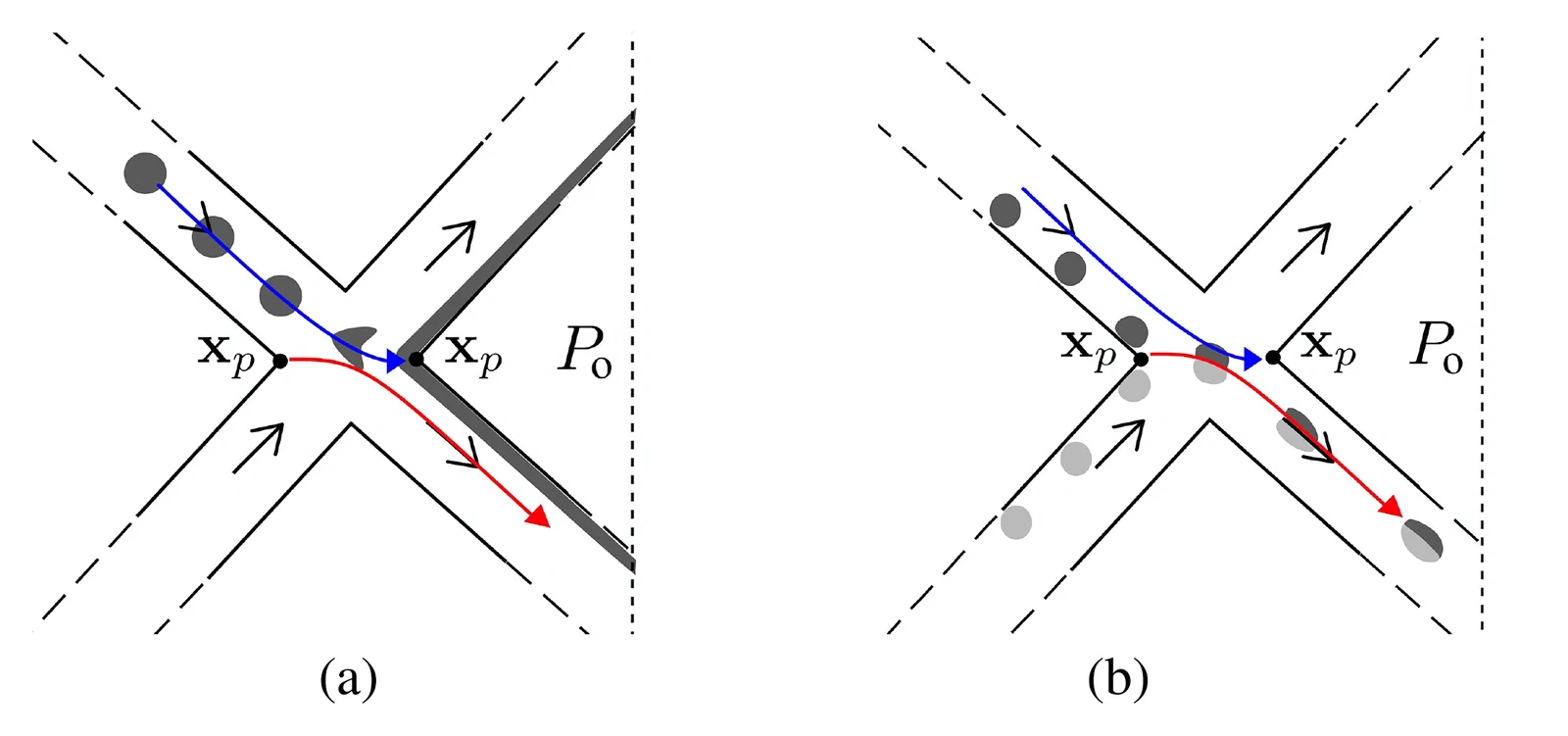
(a) Stretching and folding (SF) due to hold-up of a fluid element (shaded, shown at successive times) at the stagnation point **x**_*p*_ of a fracture intersection. As the “legs” of the fluid element are advected downstream into separate outlets, the element is stretched exponentially in time at **x**_*p*_. Further downstream, the legs of the element are effectively disconnected (“cut”) when their intersection with an arbitrary planar cross-section (vertical dashed line) *P*_*o*_ is considered. (b) Merger of two fluid elements (dark and light shading) as they approach a stagnation point **x**_*p*_ of a fracture intersection. Within the planar cross-section *P*_*o*_ these elements are essentially merged next to each other (as the width of the separating fluid decays exponentially at **x**_*p*_, effectively “shuffling” fluid elements together. In unison, the stretching and folding (SF) motions shown in (a) and (b) manifest as cutting and shuffling (CS) actions when considered in terms of *P*_*o*_.

Figure 7a illustrates how stretching and folding (SF) near a stagnation point **x**_*p*_ manifests as cutting and shuffling (CS): part of a fluid element is held up at **x**_*p*_, stretched and folded across the intersection, and then “cut” downstream as its legs disconnect in the cross-section *P*_0_. Conversely, two fluid elements passing near **x**_*p*_ (Figure 7b) are effectively merged, “shuffling” them together. Thus, the local SF dynamics project as CS in *P*_0_. The fine-scale interleaving in Figure 2c exemplifies CS at intersections, while hold-up at fracture critical points produces large-scale CS (Figure 2a). A similar mechanism occurs in porous media, though these admit both SF and CS (Lester *et al*. 2025*a*).

Although persistent exponential stretching is absent in 3D DFNs, fluctuating, non-shear stretching arises from local velocity variations. CS without deformation corresponds to *piecewise isometries* (PWIs), while CS with stretching yields *piecewise smooth* (PWS) transforms (Kreczak *et al*. 2017). In DFNs this stretching is sub-exponential and bounded, reflecting non-chaotic flow. Sections 3 and 4 show that mixing is exactly described by PWS transforms, which are quantified via a graph-based representation in the following section.

## 3 Graph Representation of Advective Mixing

To develop a graph representation of mixing, we begin by labelling the *N*_*F*_ fractures within the fracture network as *f_n_* with *n* = 1 : *N*_*F*_ and the *N*_*I*_ intersections as *I*_*n*_ with *n* = 1 : *N*_*I*_ (where *N*_*F*_ ⩽ *N*_*I*_), where, as shown in Figure 1b, inlets and outlets to the network are also considered as intersections. The number of inlet, interior and outlet intersections are respectively denoted $N_{I_{i}},N_{I_{b}},N_{I_{o}}$, such that $N_{I}=N_{I_{i}}+N_{I_{b}}+N_{I_{o}}$, and the *N*_*I*_ intersections *I*_*n*_ are ordered such that *n* = 1 : *N*_*I_i_*_ are the inlet intersections, $n=N_{I_{i}}+1:N_{I_{i}}+N_{I_{b}}$ are the interior intersections, and $n=N_{I}-N_{I_{o}}:N_{I}$ are the outlet intersections.

### 3.1 Global and Local Streamfunctions

**Figure 8:**
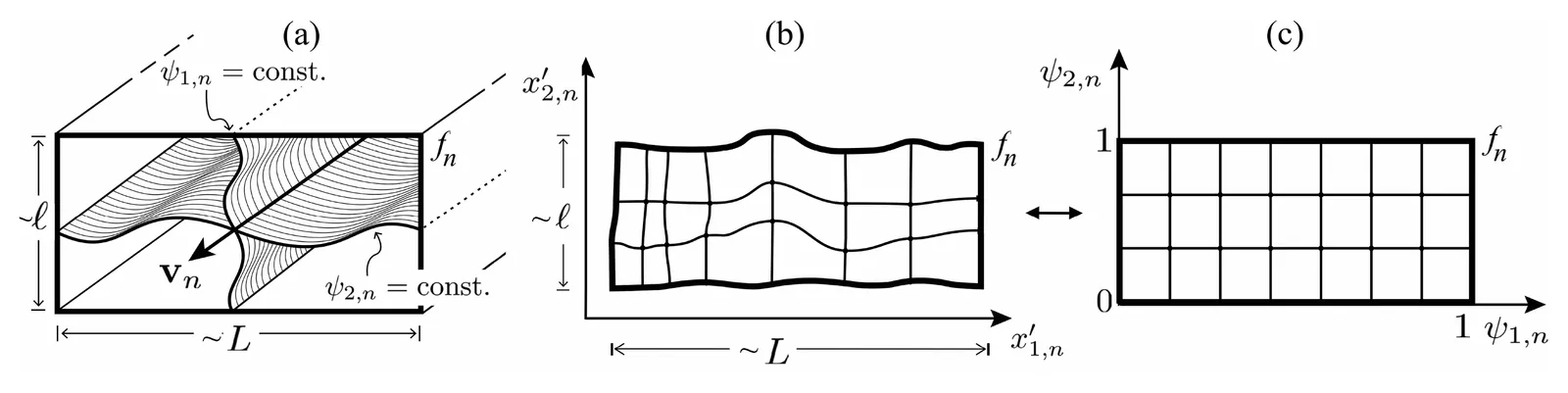
(a) Intersection of local streamfunctions (*ψ*_1,*n*_, *ψ*_2,*n*_) within the face *f_n_* of the fracture network (with dimensions ~*L* ×~𝓁) that define a streamline with local velocity **v**_*n*_. (b) Distribution of the local *ψ*_1,*n*_ (vertical lines) and *ψ*_2,*n*_ (horizontal lines) streamsurfaces over the face *f_n_* of a rough fracture of variable aperture. The mapping from local spatial coordinates $\left(x_{1,n}^{\prime },x_{2,n}^{\prime }\right)$ to local streamfunction coordinates (*ψ*_1,*n*_, *ψ*_2,*n*_) (c) generates a structured orthogonal coordinate system over face *f_n_*. Note this structured grid is also depicted throughout the intersection shown in Figure 3b.

Under this framework, the invariant streamfunctions $\left({\hat{\psi }}_{1},{\hat{\psi }}_{2}\right)\in [0,1]\times [0,1]$ in (2.3) are termed *global* streamfunctions as they uniquely label each streamline over the entire fracture network. In addition to these global streamfunctions, we also define *local* streamfunctions (*ψ*_1,*n*_, *ψ*_2,*n*_) (shown in Figure 8) over each face *f_n_* of the network (e.g. Figure 2b,d). These local streamfunctions characterise the local distribution of streamlines over *f_n_* and so facilitate quantification of advective mixing.

The local streamfunctions are also normal (*ψ*_1,*n*_, *ψ*_2,*n*_) ∈ [0, 1] × [0, 1]) and orthogonal (**∇***ψ*_1,*n*_ ·**∇***ψ*_2,*n*_ = 0) and are quantified in terms of the *local* velocity field **v**_*n*_ as

(3.1) $${\text{v}}_{n}(\text{x})\equiv \text{v}(\text{x})\;\forall \;\text{x}\in S_{m},$$

where *S*_*n*_ is the 2D surface that spans the face *f_n_*. Consistent with the dual streamfunction representation (2.3), the local streamfunctions (*ψ*_1,*n*_, *ψ*_2,*n*_) are then defined as

(3.2) $${\text{v}}_{n}(\text{x})\equiv Q_{n}\nabla \psi_{1,n}(\text{x})\times \nabla \psi_{2,n}(\text{x}),\;n=1:N_{I},\;\psi_{i,n}\in [0,1],\;i=1,2,$$

where Q_*n*_ is the volumetric flow rate through face *f_n_*. From (3.2), the face *f_n_* is bound by four stream surfaces: *ψ*_1,*n*_ = 0, *ψ*_1,*n*_ = 1, *ψ*_2,*n*_ = 0, *ψ*_2,*n*_ = 1. As **∇** *ψ*_1,*n*_ and **∇** *ψ*_2,*n*_ are both parallel to *S*_*n*_, the fluid flow rate *Q_n_* through this face is then conserved (Bear 1972)

(3.3) $$Q_{n}=\iint_{S_{n}}{\text{v}}_{n}(\text{x})\cdot d\text{S}=Q_{n}\int_{0}^{1}\int_{0}^{1}d\psi_{1,n}\;d\psi_{2,n}=Q_{n}.$$

The local streamfunctions (*ψ*_1,*n*_, *ψ*_2,*n*_) represent a local re-labelling of the global streamfunctions over face *f_n_*. For example, if the ${\hat{\psi }}_{1}$-streamsurface ${\hat{\psi }}_{1}$ = *a* = const. intersects face *f_n_*, this streamsurface also satisfies *ψ*_1,*n*_ = *b* = const., but in general *a* ≠ *b*. Hence a streamline may be labelled by the unique pair of *global* streamfunctions $\left({\hat{\psi }}_{1},{\hat{\psi }}_{2}\right)$ that are invariant over the entire network, but this streamline also traverses the fracture network via a sequence of faces *f*_*n*_1__, *f*_*n*_1__, … with *local* streamfunction values $\left(\psi_{1,n_{1}},\psi_{2,n_{1}}\right),\left(\psi_{1,n_{2}},\psi_{2,n_{2}}\right),\ldots$. As such, the global streamfunctions $\left({\hat{\psi }}_{1},{\hat{\psi }}_{2}\right)$ are used to *label* a given streamline while the local streamfunctions (*ψ*_1,*n*_, *ψ*_2,*n*_) are used to characterise the *local position* of that streamline in face *f_n_*. Hence advective mixing can be quantified by the evolution of (*f_n_*, *ψ*_1,*n*_, *ψ*_2,*n*_) for a given streamline.

As illustrated in Figure 8, *ψ*_1,*n*_ varies along the fracture width (~*L*) of face *f_n_* and *ψ*_2,*n*_ varies along the fracture aperture (~𝓁), with streamlines arising at the intersection of local *ψ*_1,*n*_- and *ψ*_2,*n*_-streamsurfaces. We also introduce the local Eulerian spatial coordinates $\left(x_{1,n}^{\prime },x_{2,n}^{\prime }\right)\in [0,\sim L]\times [0,\sim \ell ]$ (Figure 8b) that span *f_n_* and their dimensionless counterparts $\left(x_{1,n},x_{2,n}\right)\in [0,1]\times [0,1]$. Figure 8c shows that the mapping from local spatial coordinates $\left(x_{1,n}^{\prime },x_{2,n}^{\prime }\right)$ to local streamfunction coordinates $\left(\psi_{1,n},\psi_{2,n}\right)$ allows rough fractures with variable aperture to be mapped onto a structured orthogonal grid. As shown in Figure 3b, this means that even if rough, 3D intersections are spanned and bounded by a structured orthogonal grid of ${\hat{\psi }}_{1}$ - and ${\hat{\psi }}_{2}$-streamsurfaces, where the ${\hat{\psi }}_{1}$-streamsurfaces are coherent 2D cross-sections of the intersection.

For fracture networks with only one inlet fracture (i.e $N_{I_{i}}=1$), the outlet face (where fluid enters the network) is defined as $f_{n}=f_{1}^{o}$ with flow rate $Q_{1}^{o}$. Hence the total network flow rate is $Q=Q_{1}^{o}$, and from (2.3), (3.2) we can also define the global and local streamfunctions to coincide over face *f_n_* as

(3.4) $$\psi_{1,n=1}\equiv {\hat{\psi }}_{1}\;\psi_{2,n=1}\equiv {\hat{\psi }}_{2}.$$

Conversely, for fracture networks with multiple inlet fractures (*N_I_i__* > 1), the corresponding outlet faces can be defined as $f_{n}=f_{n}^{o}$ with *n* = 1 : *I*_*i*_, and the total network flow rate *Q* is

(3.5) $$Q=\sum_{n=1}^{N_{I_{i}}}Q_{n}^{o},$$

where $Q_{n,1}^{o}$ is the total flowrate through face $f_{n}^{o}$. From (2.3), (3.2), we can uniquely define the global streamfunctions by spanning the ${\hat{\psi }}_{1}$ streamfunction over the $N_{I_{i}}$ ordered inlets as

(3.6) $$\psi_{1,m}\equiv \frac{1}{Q_{m,1}^{o}}\left(Q{\hat{\psi }}_{1}-\sum_{n=1}^{m-1}Q_{n,1}^{o}\right)\;\psi_{2,m}\equiv {\hat{\psi }}_{2},\;m\in \left[1:N_{I_{i}}\right]$$

where *m* is the index of the outlet face $f_{m}^{o}$ corresponding to $\psi_{1,m},{\hat{\psi }}_{1}$. Note the flow rates in (3.6) arise from normalisation of the streamfunctions in (2.3), (3.2). The local streamfunctions $\left(\psi_{1,n},\psi_{2,n}\right)$ for the remaining faces (i.e. *n* > *N_I_i__*) in the network are defined by (3.2). Together, the local and global streamfunctions can be used to quantify intersection and fracture mixing.

### 3.2 Intersection and Fracture Mixing Maps

We first develop a method to route streamlines between faces in the network. As a streamline is advected from face *f*_*j*_ (with local streamfunctions *ψ*_1, *j*_, *ψ*_2, *j*_) in the network, the next face *f*_*k*_ it encounters (and local streamfunctions *ψ*_1,*k*_, *ψ*_2,*k*_) is determined by streamline routing. The relationship between these faces and local streamfunctions can be efficiently encoded via two classes of algebraic maps we term *mixing maps*. If face *f*_*j*_ is an inlet face, the streamline then passes through an intersection and (*f*_*k*_, *ψ*_1,*k*_, *ψ*_2,*k*_) is determined by an *intersection mixing map* ***ℳ_I_***. Conversely, if face *f*_*j*_ is an outlet face, the streamline then passes through a fracture and (*f*_*k*_, *ψ*_1,*k*_, *ψ*_2,*k*_) is determined by a *fracture mixing map* ***ℳ_F_***. As streamlines are routed through either intersections or fractures with fixed global streamfunctions $\left({\hat{\psi }}_{1},{\hat{\psi }}_{2}\right)$, the relationship between the local streamfunctions at faces *f*_*j*_ and *f*_*k*_ is given by the mappings between the global and local streamfunctions at these faces.

For the case of intersection mixing, we show in Appendix B that if the spatial distribution of areal fluxes

(3.7) $$q_{n}\left(x_{1}\right)\equiv \int_{0}^{1}\|{\text{v}}_{n}\|d\psi_{2,n}=Q_{n}\nabla \psi_{1,n},$$

between the inlet face *f*_*j*_ and outlet face *f*_*k*_ of an intersection are proportional, i.e. *q*_*j*_ (*x*_1_) / *q*_*k*_ (*x*_1_) = const., then the local *ψ*_1_ streamfunctions are equivalent, i.e. *ψ*_1,*k*_ = *ψ*_1, *j*_. Hence streamline routing only alters the *ψ*_2_ coordinate. This condition renders intersection mixing a PWI transform, where fluid elements are cut and shuffled with respect to *ψ*_2_ without stretching in the *ψ*_1_ direction. As such, the local streamfunctions evolve according to

(3.8) $$\left(\psi_{2,k},f_{k}\right)={\text{ℳ}}_{I}\left(\psi_{2,j},f_{j}\right)\;\psi_{1,k}=\psi_{1,j}.$$

If the flux condition is violated, i.e. *q*_*j*_ (*x*_1_) / *q*_*k*_ (*x*_1_) ≠ const., then intersection mixing is a PWS transform, where fluid stretching along the *ψ*_1_ direction can occur in conjunction with cutting and shuffling with respect to *ψ*_2_. In this case the intersection mixing map ***ℳ_I_*** acts on both local streamfunctions as

(3.9) $$\left(\psi_{1,k},\psi_{2,k},f_{k}\right)={\text{ℳ}}_{\text{I}}\left(\psi_{1,j},\psi_{2,j},f_{j}\right).$$

We refer to (3.8) and (3.9) respectively as the PWI and PWS representations of ***ℳ_I_***, where the PWI map is significantly simpler in that it can be completely quantified solely in terms of the total fluxes *Q_n_* through faces, whereas the PWS map requires the areal fluxes *q_n_*(*x*_1_) at each face of the intersection (see Appendix B for details). Throughout §2-3 we use ***ℳ_I_*** arbitrarily to denote either the PWI or PWS maps, and in §4 we will directly test each representation by comparison against numerical simulation.

During fracture mixing, streamlines flow from the outlet face *f*_*k*_ to the inlet face *f*_*l*_. The large aspect ratio 𝓁 ≪ *L* renders the variation between *ψ*_2, *j*_ and *ψ*_2,*k*_ negligible (𝒪 (𝓁/ *L*)), and so only the local *ψ*_1_ streamfunction is significantly altered

(3.10) $$\left(\psi_{1,l},f_{l}\right)={ℳ}_{F}\left(\psi_{1,k},f_{k}\right)\;\psi_{2,l}=\psi_{2,k}.$$

For a given streamline, the overall map **ℳ** which quantifies the evolution of local streamfunctions through the fracture network is given by a sequential composition of ***ℳ_F_***, ***ℳ_I_*** as

(3.11) $$ℳ={\text{ℳ}}_{F}\circ {\text{ℳ}}_{I}\circ \ldots {\text{ℳ}}_{F}\circ {\text{ℳ}}_{I},$$

reflecting that streamlines sequentially travel through fractures and intersections. Hence the maps ***ℳ_I_***, ***ℳ_F_*** encode properties of the fracture network that govern the streamline routing process. Note that **ℳ**_*F*_, ***ℳ_I_*** are *local* in that they are specific to each face *f_n_*. From (3.11), the routing of a given streamline is characterised by the sequence of local streamfunctions and faces $f_{n_{j}}$ for *j* = 1, …, *N* it traverses through the fracture network

(3.12) $$\left\{\left(\psi_{1,f_{n_{1}}},\psi_{2,f_{n_{1}}},f_{n_{1}}\right),\left(\psi_{1,f_{n_{2}}},\psi_{2,f_{n_{2}}},f_{n_{2}}\right),\ldots ,\left(\psi_{1,f_{n_{N}}},\psi_{2,f_{n_{N}}},f_{n_{N}}\right)\right\},$$

where

(3.13) $$\left(\psi_{1,f_{n_{i}}},\psi_{2,f_{n_{i}}},f_{n_{i}}\right)={\text{ℳ}}_{F}\circ {\text{ℳ}}_{I}\circ \ldots {\text{ℳ}}_{F}\circ {\text{ℳ}}_{I}\left(\psi_{1,f_{n_{1}}},\psi_{2,f_{n_{1}}},f_{n_{1}}\right).$$

This characterisation of local streamfunctions (*ψ*_1,*n*_, *ψ*_2,*n*_) and faces *f_n_* completely quantifies advective mixing within the fracture network. Algebraic expressions for ***ℳ_I_***, ***ℳ_F_*** based on respective flux balances within intersections and fractures are derived in Appendix B, and in the following subsection they are embedded in an efficient graph representation of mixing.

### 3.3 Intersection Graph *𝒢_I_*

Previous studies have used graph representations to quantify flow and transport in DFNs, via both fracture graphs ***𝒢_F_*** (where vertices represent fractures and edges represent intersections) and intersection graphs (vice-versa) ***𝒢_I_***. To accommodate the mixing mechanisms identified in §2, we extend the intersection graph ***𝒢_I_*** to form the mixing graph ***𝒢_M_***. The intersection graph ***𝒢**_I_* is defined by the set of *N*_*I*_ vertices given by the intersections *I*_*m*_ and the set of *N*_*e*_ graph edges *e*_*n*_ (with weights *Q*_*n*_) given by flows between intersections within each fracture, hence ***𝒢_I_*** is a weighted acyclic directed graph (WADG). Here we use modified nomenclature where the *n*-th inlet (outlet) face of intersection *I*_*m*_ is denoted $f_{m,n}^{i},n=1:N_{I_{i}}$ with flow rate $Q_{m,n}^{i}$ ($f_{m,n}^{o},n=1:NI_{o}$ with flow rate $Q_{m,n}^{o}$) where $N_{m}^{i}\in [1,3]\left(N_{m}^{o}\in [1,3]\right)$ is the total number of inlet (outlet) faces of intersection *I*_*m*_. Using this nomenclature, all interior intersections *I*_*m*_ satisfy flow conservation

(3.14) $$Q_{m}=\sum_{n=1}^{N_{m}^{i}}Q_{m,n}^{i}=\sum_{n=1}^{N_{m}^{o}}Q_{m,n}^{o}\;\text{for}\;m=N_{I_{i}}+1:N_{I}-N_{I_{o}}.$$

Note that each inlet into the overall fracture network is considered to be an intersection *I*_*m*_ with one outlet face $\left(N_{m}^{o}=1\right)$, and each outlet is considered to be an intersection *I*_*m*_ with only one inlet face $\left(N_{m}^{i}=1\right)$. From (3.5), the total flow rate through the network is

(3.15) $$Q=\sum_{m=1}^{N_{I_{i}}}Q_{m,1}^{o}=\sum_{m=N_{I}-N_{I_{o}}}^{N_{I}}Q_{m,1}^{i}.$$

**Figure 9:**
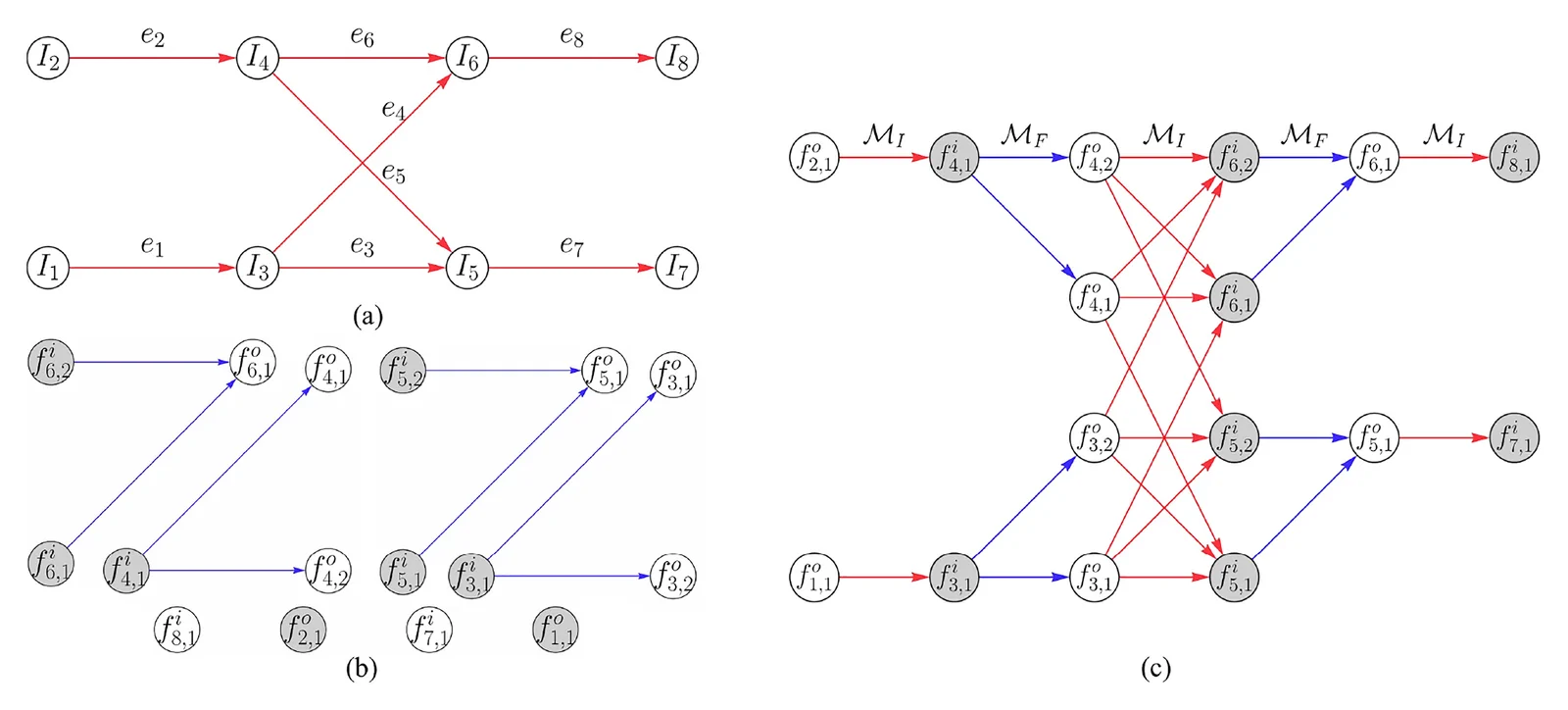
(a) Intersection graph ***𝒢_I_*** of a simple DFN depicting intersections *I*_*m*_ (white vertices) and connecting flows as directed edges *e*_*n*_ (red arrows). (b) Expansion of intersections *I*_*m*_ in (a) into intersection clusters *c*_*m*_ comprised of inlet faces $f_{m,n}^{i}$ (grey vertices) and outlet faces $f_{m,n}^{o}$ (white vertices) and connecting flows as directed edges (blue arrows). (c) Mixing graph ***𝒢_M_*** formed by replacing the *N*_*I*_ intersection vertices *I*_*m*_ of ***𝒢_I_*** shown in (a) with the clusters *c*_*m*_ shown in (b) and connecting the inlet faces $f_{m,n}^{i}$ (grey vertices) and outlet faces $f_{m,n}^{o}$ (white vertices) via streamline connections within fractures. Red arrows depict connection of faces via intersections and streamline routing via the intersection map ***ℳ_I_***, and blue arrows depict connection of faces via fractures and streamline routing via the intersection map ***ℳ_F_***.

These properties mean that the intersection graph ***𝒢_I_*** may also be classified as a zero-capacity *flow network* (Ahuja *et al*. 1993). Figure 9a shows the intersection graph ***𝒢_I_*** for the simple DFN shown in Figure 1b which is comprised of *N*_*F*_ = 5 fractures and *N*_*I*_ = 8 intersections, which comprise *N*_*I*,*i*_ = 2 inlet intersections, $N_{I_{b}}=4$ interior intersections and $N_{I_{0}}=2$ outlet intersections.

### 3.4 Mixing Graph *𝒢_M_*

As the intersection graph ***𝒢_I_*** does not resolve the intersection faces *f_n_*, it must be extended to form the mixing graph ***𝒢_M_***. Each intersection *I*_*m*_ is accompanied by $N_{m}^{i}$ inlet faces $f_{m,n}^{i}$ ordered in the direction of increasing *ψ*_2,*n*_ as $n=1:N_{m}^{i}$, and $N_{m}^{o}$ outlet faces $f_{m,n}^{o}$ ordered in the same manner as $n=1:N_{m}^{o}$. Hence an *intersection cluster C*_*m*_ can be formed for each intersection *I*_*m*_ (shown in Figure 9b) that is comprised of the inlet faces $f_{m,n}^{i}$ and outlet faces $f_{m,n}^{o}$ connected by *I*_*m*_. ***𝒢_M_*** is then formed by replacing each of the intersection vertices *I*_*m*_ in ***𝒢_I_*** with its intersection cluster *C*_*m*_. Note that the inlet (*I*_1_, *I*_2_) and outlet intersections (*I*_7_, *I*_8_) in Figure 9b only contain a single outlet face $f_{m,n}^{o}$ or inlet face $f_{m,n}^{i}$, and so their intersection clusters *C*_*m*_ are comprised of a single vertex. The directed graph edges between inlet $f_{m,n}^{i}$ and outlet $f_{m,n}^{o}$ faces (blue arrows in Figure 9) are termed *intersection edges* as they are associated with flow through intersections, and the directed edges between outlet $f_{m,n}^{o}$ and inlet $f_{m,n}^{i}$ faces (red arrows in Figure 9) are termed *fracture edges* and are associated with flow through fractures. Similar to ***𝒢_I_***, ***𝒢_M_*** is a WADG and a flow network.

**Figure 10:**
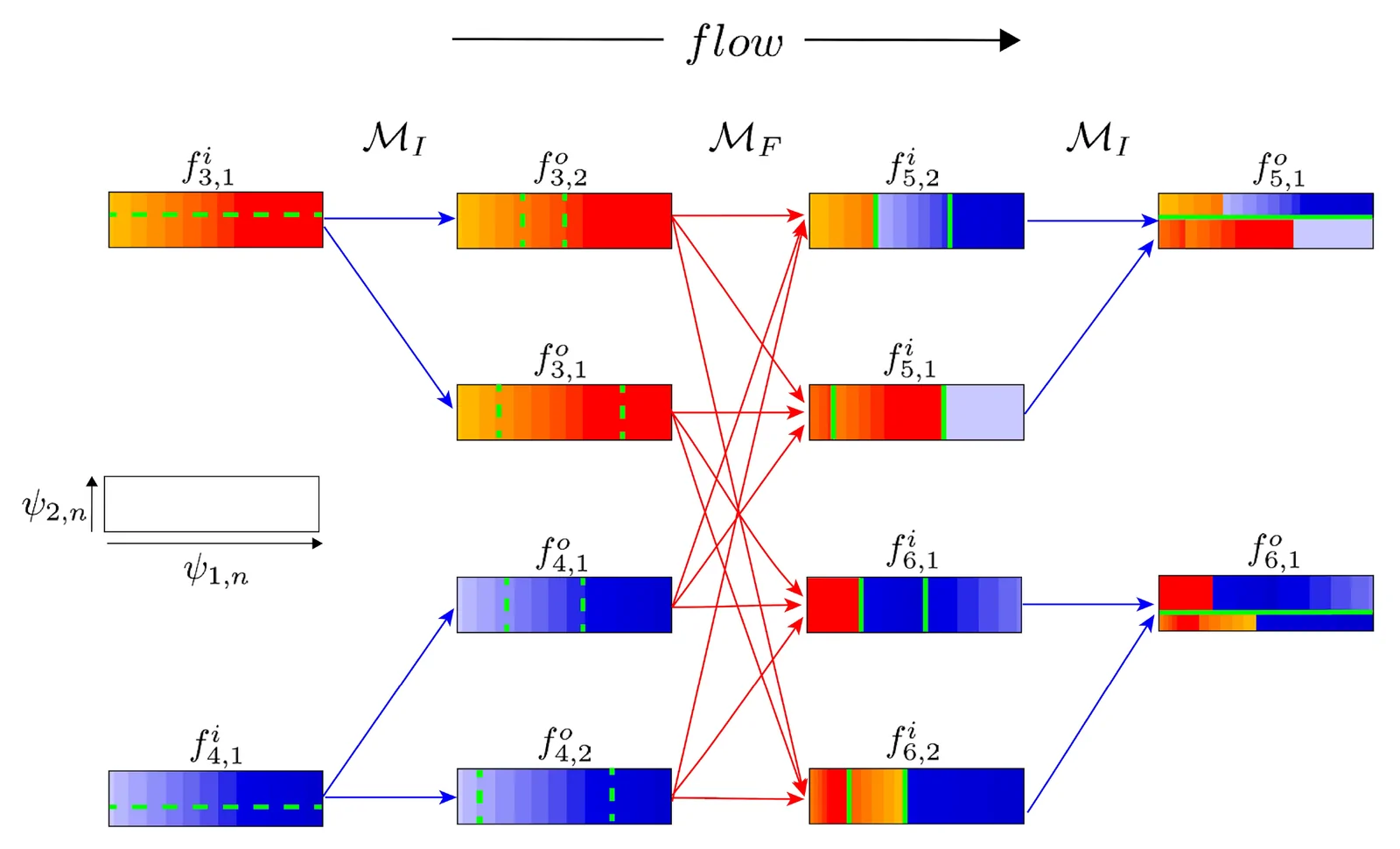
Schematic of the evolution of the 2D concentration profile *c*_*n*_ (*ψ*_1,*n*_, *ψ*_2,*n*_) (coloured rectangles) at each face *f_n_* of the fracture network shown in Figure 1b, represented in terms of the mixing graph ***𝒢_M_*** shown in Figure 9c. As shown, the inlet concentration field at the fracture inlets $f_{3,1}^{o}\;$ and $f_{4,1}^{o}$ comprises of red and blue coloured segments that only vary in the *ψ*_1_ direction. Dashed green horizontal (vertical) lines represent splitting or “cutting” due to intersection (fracture) mixing, and solid green horizontal (vertical) lines represent merging or “shuffling” due to intersection (fracture) mixing.

As shown in Figure 9c, streamlines are advected through the graph vertices via the periodic sequence (*outlet face, inlet face, outlet face*, …). The intersection maps ***𝒢_I_*** and fracture maps ***𝒢_F_*** respectively apply at the intersection and fracture edges of the graph, forming a dynamic process known as a *sequential dynamical system* (SDS) (Mortveit & Reidys 2007), a class of discrete dynamical system embedded in graph structures such as ***𝒢_M_***. Here the topology of the SDS (fracture network) is encoded by ***𝒢_M_***, and the dynamics (streamline routing) are encoded by the maps ***ℳ_I_***, ***ℳ_F_*** respectively acting at intersection edges and fracture edges. The evolution of a scalar concentration field under the action of ***𝒢_M_*** is shown in Figure 10, which highlights the CS actions that control mixing.

### 3.5 Quantification of G_M_ and Computational Overhead

The information required to construct the mixing graph ***𝒢_M_*** under the PWI or PWS models is summarised as follows. First, the fractures and intersections of the network must be identified as well as the intersection graph ***𝒢_I_*** (Figure 9a), including identification of dead-end fractures and splitting intersections into “inflow only” or “outflow only” regions with respect to the dead-end fracture (see§§ 2.4 for details). For each intersection, the relevant faces *f_n_* must be identified along with their volumetric flow rates *Q_n_* and the relative ordering of these faces around the intersection as detailed in Appendix B. This information is sufficient to quantify the intersection mixing map ***ℳ_I_*** under the PWI model. For the PWS model, the areal fluxes *q_n_*(*x*_1_) along the faces are also required to quantify ***ℳ_I_***. For the fracture mixing map ***ℳ_F_***, the separating streamlines in each fracture must be identified, along with the streamline topology (Figure 1c) that routes streamlines from face to face within the fracture and the associated fluxes *Q*_*j*,*n*_, as detailed in Appendix B. This topological information allows construction of the clusters *C*_*m*_ associated with each intersection (Figure 9b), facilitating construction of the mixing graph ***𝒢_M_*** (Figure 9b) and quantification of advective mixing via the maps ***ℳ_I_***, ***ℳ_F_***.

Hence the information required to quantify the PWI (PWS) model can be readily determined from DFN models by identifying all faces *f_n_* and computing the flow rates *Q_n_* (*q_n_* (*x*_1_)), and computing the separating streamlines within the fractures and the associated fluxes *Q*_*j*,*n*_ between these streamlines. As shown in Figure 1c, these separating streamlines can be identified and constructed by integrating tracer streamlines forward and backward in time from stagnation points and the end points of intersections. Hence the mixing graph ***𝒢_M_*** can be constructed from minimal information from the DFN, yet resolves the complex advective mixing dynamics with minimal computational overhead. This information (distribution of fluxes, streamline topology, etc) can also be characterised in a statistical sense, facilitating development of stochastic models of advective mixing in large random DFNs.

Streamline routing fracture networks is computed efficiently via ***𝒢_M_***. For a given streamline, we denote *n*_*F*_, *n*_*I*_ = *n*_*F*_ + 1 respectively as the number of fractures and intersections routed from network inlet to outlet. From the operations in Appendix B, the number of floating point operations (FLOPS) for the fracture map ***ℳ_F_*** is *n*_*o*_ +*n*_*i*_ + 2. For the intersection map ***ℳ_I_***, the PWI model also incurs *n*_*o*_ +*n*_*i*_ +2 FLOPS, whereas the PWS model incurs *n*_*o*_+ *n*_*i*_ +4 function valuations (*q*_*n*_ (*x*_1_), Ψ_1,*n*_ (*x*_1_), *X*_1,*n*_ (*ψ*_1_)) of *p*^2^ FLOPS each if they involve order *p* spline functions. Thus this advection of *N* streamlines requires *N* (⟨*n*_*F*_ ⟩ + *p*^2^ ⟨*n*_*I*_ ⟩) (⟨*n*_*o*_ ⟩ + ⟨*n*_*i*_ ⟩ + 1) FLOPS, where ⟨*n*⟩ denotes the average of *n* and *p* = 1 for the PWI model.

#### 3.6 Scalar Transport and Mixing Measures

To quantify advective mixing in 3D DFNs, we consider mixing of a non-diffusive scalar with a steady concentration distribution field *c* that is defined in terms of the inlet concentration distribution (i.e. over the network inlets) as $c_{0}\left({\hat{\psi }}_{1},{\hat{\psi }}_{2}\right)$. For simplicity of exposition, we consider the inlet distribution $c_{0}\left({\hat{\psi }}_{1},{\hat{\psi }}_{2}\right)=H\left(1/2-{\hat{\psi }}_{1}\right)$ (where *H* is the Heaviside step function), but note that the complete mixing properties of the fracture network depends upon the mixing characteristics across all possible *c*_0_. The local solute concentration distribution *c*_*n*_ (*ψ*_1,*n*_, *ψ*_2,*n*_) at each inlet face $f_{n},n=1:N_{I_{i}}$ of the fracture network may be given in terms of the local streamfunctions as

(3.16) $$c_{n}\left(\psi_{1,n},\psi_{2,n}\right)\equiv c_{0}\left[{\hat{\psi }}_{1}\left(\psi_{1,n},n\right),{\hat{\psi }}_{2}\left(\psi_{2,n},n\right)\right],\;n=1:N_{I_{i}}.$$

As the scalar concentration is invariant along streamlines, then for all faces

(3.17) $$c_{n}\left(\psi_{1,n},\psi_{2,n}\right)=c_{0}\left[{\hat{\psi }}_{1}\left(\psi_{1,n},\psi_{2,n},n\right),{\hat{\psi }}_{2}\left(\psi_{1,n},\psi_{2,n},n\right)\right],\;n=1:N_{I}.$$

Figure 10 shows evolution of *c*_*n*_ across the intersection faces *f_n_* for the simple fracture network shown in Figure 1b (under the PWI model for ***ℳ_I_***). This figure illustrates how advective mixing acts to cut and shuffle the local concentration field *c*_*n*_ in both the *ψ*_1,*n*_ and *ψ*_2,*n*_ directions via the respective fracture (***ℳ_F_***) and intersection (***ℳ_I_***) mixing mechanisms.

To examine mixing over the entire fracture network, it is useful to construct the outlet concentration distribution *c*_out_ as the aggregated concentration distribution over the network outlets. To do so, we aggregate all inlet faces $f_{m,1}^{i}$ of the outlet intersections *I*_*m*_ with $m=N_{I}-N_{I_{o}}+1:N_{I}$ by defining a *global outlet* streamfunction ${\hat{\psi }}_{1}^{\text{out}\;}\in [0,1]$ that concatenates the local *ψ*_1_ streamfunctions *ψ*_1,*m*_ weighted by their respective fluxes *Q*_*m*_ as

(3.18) $${\hat{\psi }}_{1}^{\text{out}}\equiv \frac{1}{Q}\left(\sum_{j=N_{I}-N_{I_{o}}+1}^{m-1}Q_{j}+Q_{m}\psi_{1,m}\right),\;m=N_{I}-N_{I_{o}}+1:N_{I}.$$

As these outlets are concatenated along the *ψ*_1_ coordinate, the global *ψ*_2_ streamfunction at the outlet is simply ${\hat{\psi }}_{2}^{\text{out}\;}=\psi_{2,m}$. Hence the outlet global streamfunctions can be expressed in terms of the global streamfunctions as

(3.19) $${\hat{\psi }}_{1}^{\text{out}\;}={\hat{\psi }}_{1}^{\text{out}\;}\left({\hat{\psi }}_{1},{\hat{\psi }}_{2}\right)\;{\hat{\psi }}_{2}^{\text{out}\;}={\hat{\psi }}_{2}^{\text{out}\;}\left({\hat{\psi }}_{1},{\hat{\psi }}_{2}\right).$$

**Figure 11:**
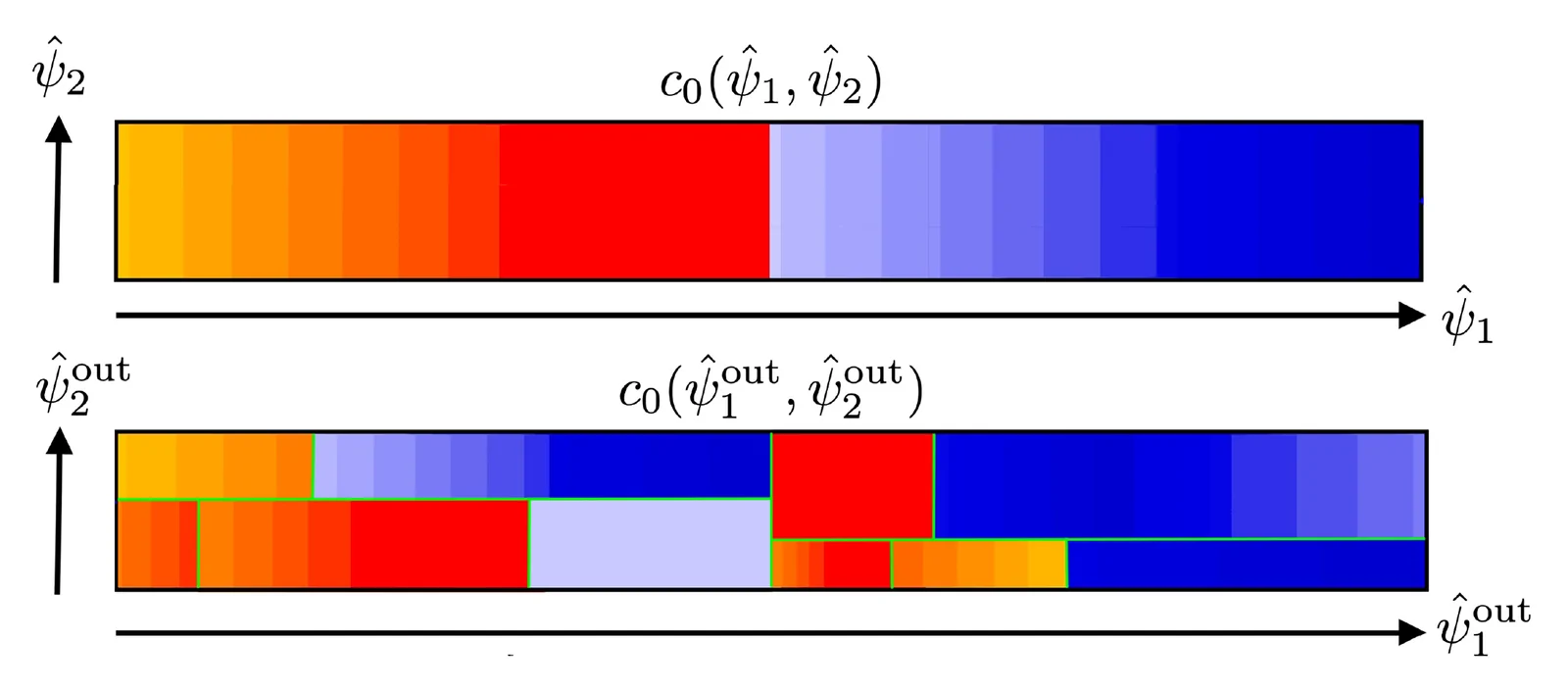
Mapping of the inlet concentration $c_{0}\left({\hat{\psi }}_{1},{\hat{\psi }}_{2}\right)$ shown in Figure 10 to the outlet concentration $c_{\text{out}\;}\left({\hat{\psi }}_{1},{\hat{\psi }}_{2}\right)\equiv c_{0}\left({\hat{\psi }}_{1}^{\text{out}\;},{\hat{\psi }}_{2}^{\text{out}\;}\right)$. The resultant concentration profile is a direct result of the CS actions mediated by the separating streamsurfaces (indicated by green dashed lines) in the DFN.

The outlet concentration profile *c*_out_ is then

(3.20) $$c_{\text{out}\;}\left({\hat{\psi }}_{1},{\hat{\psi }}_{2}\right)\equiv c_{0}\left[{\hat{\psi }}_{1}^{\text{out}\;}\left({\hat{\psi }}_{1},{\hat{\psi }}_{2}\right),{\hat{\psi }}_{2}^{\text{out}\;}\left({\hat{\psi }}_{1},{\hat{\psi }}_{2}\right)\right].$$

which provides a convenient basis for quantification of mixing over the entire fracture network. Figure 11 shows how the inlet concentration $c_{0}\left({\hat{\psi }}_{1},{\hat{\psi }}_{2}\right)$ is mapped to the outlet concentration $c_{0}\left({\hat{\psi }}_{1}^{\text{out}\;},{\hat{\psi }}_{2}^{\text{out}\;}\right)$ for the concentration field shown in Figure 10.

In Appendix C we show that under purely advective (non-dissipative) mixing, typical mixing measures such as concentration variance do not alter between *c*_0_ and *c*_out_. Instead we use a multi-scale mixing measure, the (squared) *mix-norm* ${\text{Φ}}_{2\text{D}}^{2}$ (Mathew *et al*. 2005), which robustly measures the degree of mixing by integrating over the scale-dependent concentration variance *ϕ*^2^ (*c*_out_, *s*) as

(3.21) $${\text{Φ}}_{2\text{D}}^{2}\equiv \frac{1}{L}\int_{0}^{L}\phi_{2\text{D}}^{2}\left(c_{\text{out}},s\right)ds,$$

see Appendix C for details.

The 2D concentration field $c_{\text{out}\;}\left({\hat{\psi }}_{1},{\hat{\psi }}_{2}\right)$ and mixing measures $\phi_{2\text{D}}^{2},{\text{Φ}}_{2\text{D}}^{2}$ provide a complete quantification of advective mixing over the fracture network. In Appendix C we also show that a coarse-grained 1D $\left(\;{\hat{\psi }}_{1}-\text{only}\right)$ representation also provides a highly accurate representation of advective mixing for 𝓁 ≪ *L*. In some instances, this coarse-grained representation is preferable to simplify visualization of mixing and connect with observable concentration fields. Following Appendix C, a simple coarse-graining approach is employed which consists of averaging *c*_0_ and *c*_out_ with respect to ${\hat{\psi }}_{2}$ to yield

(3.22) $$c\left({\hat{\psi }}_{1}\right)\equiv \int_{0}^{L}c\left({\hat{\psi }}_{1},{\hat{\psi }}_{2}\right)d{\hat{\psi }}_{2},\;c=c_{0},c_{\text{out}},$$

where $c\left({\hat{\psi }}_{1}\right)$ is differentiated from $c\left({\hat{\psi }}_{1},{\hat{\psi }}_{2}\right)$ by its argument. Similarly, the global streamfunctions can be coarse-grained as ${\hat{\psi }}_{2}^{\text{out}\;}$) ↦ ${\hat{\psi }}_{1}^{\text{out}\;}$. Note that this averaging yields a simplified *representation* of advective mixing rather than a physical mixing process. The associated 1D scale-dependent variance $\phi_{1\text{D}}^{2}(s)$ and mix-norm ${\text{Φ}}_{1\text{D}}^{2}$ are defined analogously as their respective 2D counterparts in (C 5), (C 6), and are related to the 2D measures as

(3.23) $$\phi_{1\text{D}}^{2}(s)=\{\begin{array}{ll} 0 & 0<s⩽\ell ,\\ \phi_{2\text{D}}^{2}(s) & \ell <s⩽L, \end{array}\;{\text{Φ}}_{1\text{D}}^{2}={\text{Φ}}_{2\text{D},L}^{2}.$$

Hence the 1D representation provides an accurate representation of mixing dynamics and the associated 1D mixing measures $\phi_{1\text{D}}^{2},{\text{Φ}}_{1\text{D}}^{2}$ provide accurate estimates of their 2D counterparts $\phi_{\text{2D}}^{2},{\text{Φ}}_{\text{2D}}^{2}$.

Many 2D DFN models perform similar averaging on physical grounds, based on the assumption of complete mixing at fracture intersections. The validity of this assumption is quantified the two Péclet numbers associated with the *ψ*_1_ and *ψ*_2_ coordinates, defined as

(3.24) $$Pe_{\|}\equiv \frac{v_{c}L}{D_{m}}\;Pe_{⊥}\equiv \frac{v_{c}\ell }{D_{m}},$$

where *v*_*c*_ is the characteristic velocity scale and *D*_*m*_ is the diffusion coefficient. Under such models, solute mixing is considered to be advection-dominated in the *ψ*_1_ direction $\left(Pe_{\|}\gg 1\right)$ and diffusion-dominated in the *ψ*_2_ direction $\left(Pe_{⊥}\ll 1\right)$, justifying the “well-mixed” assumption commonly evoked at fracture intersections (Hyman & Jiménez-Martínez 2018). However such models cannot be readily generalized beyond these limits. Note that the averaging process (3.22) is not equivalent to such physical diffusion as it is applied as a post-processing step to the outlet concentration distribution *c*_out_ rather than a continual diffusion process throughout the fracture network.

## 4 Numerical Simulations

### 4.1 Test DFNs and Numerical Method

**Figure 12:**
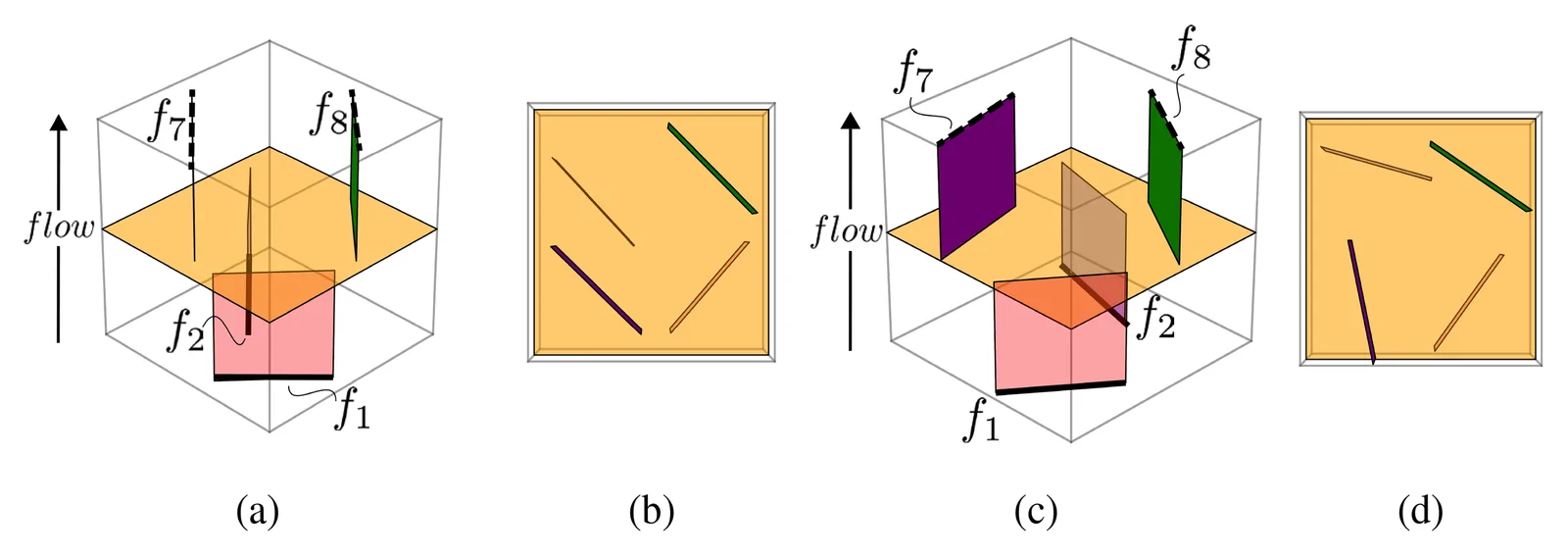
Isometric (a,c) and top (b,d) views of test DFN *A* (a,b) and DFN *B* (c,d).

The mixing measures $c_{\text{out}\;},{\text{Φ}}_{1\text{D}}^{2},\phi_{1\text{D}}^{2}(s)$ used to validate the advective mixing theory and associated mixing graph ***𝒢_M_*** by comparison with numerical simulations performed via the 2D DFN simulation code DFN.lab (Le Goc *et al*. 2019). We consider two different test DFNs and test ***𝒢_M_*** under both the PWI (3.8) and PWS (3.9) models of the intersection map ***ℳ_I_***. The first DFN considered (denoted DFN *A*) is shown in Figure 12(a,b) and corresponds to the DFN shown in Figure 1(b), which is less disordered than the second DFN considered (denoted DFN *B*) shown in Figure 12 (c,d). For both DFNs, flow in each fracture is modelled as a steady planar 2D Darcy flow with uniform transmissivity. A prescribed pressure differential between the DFN inlets and outlets fixes the total volumetric flow at *Q* = 1. The inlet concentration is set to $c_{0}\left({\hat{\psi }}_{1},{\hat{\psi }}_{2}\right)=0$ in one inlet fracture and $c_{0}\left({\hat{\psi }}_{1},{\hat{\psi }}_{2}\right)=1$ in the other. The two outlets collect the discharge and all other boundaries are no-flux (see Supplementary Material for further details). DFN.lab is used to solve the flow and advect approximately 10^6^ streamlines that are seeded along the network inlets in a flux-weighted manner.

### 4.2 Construction of Mixing Graph ***𝒢_M_***

From these numerical results, the topology of the mixing graph ***𝒢_M_*** is then determined by the connectivity (via streamlines) between fractures and intersections, and the edge weights of ***𝒢_M_*** are determined by the flow rates *Q_n_* between faces. The intersection ***ℳ_I_*** and fracture ***ℳ_F_*** maps that operate respectively on intersection edges and fracture edges are determined from streamline routing. Note that for the PWI model (3.8), the intersection maps ***ℳ_I_*** are determined solely from streamline routing data and the local *ψ*_1_ streamfunction is invariant over an intersection. Conversely, whereas *ψ*_1_ can vary under the PWS model (3.10), as is quantified by the flux distribution *q*_*n*_ over face *f_n_* as detailed in Appendix B. 2D DFN models such as DFN.lab do not resolve the *ψ*_2_ coordinate but instead perform random flux-weighted streamline routing at intersections, which is physically consistent with well-mixed conditions in the *ψ*_2_ coordinate and the 1D mixing representation outlined in Appendix C. Conversely, the PWI and PWS models for ***𝒢_M_*** perform completely deterministic streamline routing based on the mixing maps ***ℳ_I_***, ***ℳ_F_***. We note that while the methods detailed in §3 allow both *ψ*_1_ and *ψ*_2_ to be resolved, in this section we only compare 1D mixing results between DFN.lab and the mixing graph ***𝒢_M_***.

Development and application of the mixing graph ***𝒢_M_*** and the mixing maps ***ℳ_I_*** (PWI model) and ***ℳ_F_*** for DFN *A* results in the 2D concentration fields shown in Figure 10. This clearly illustrates the impact of intersection and fracture mixing mechanisms upon the local concentration field *c*_*n*_ (*ψ*_1,*n*_, *ψ*_2,*n*_) at each face *f_n_*, and the corresponding 1D mixing plot is shown in Figure 18d.

### 4.3 Comparison of Advective Mixing in Test DFNs

**Figure 13:**
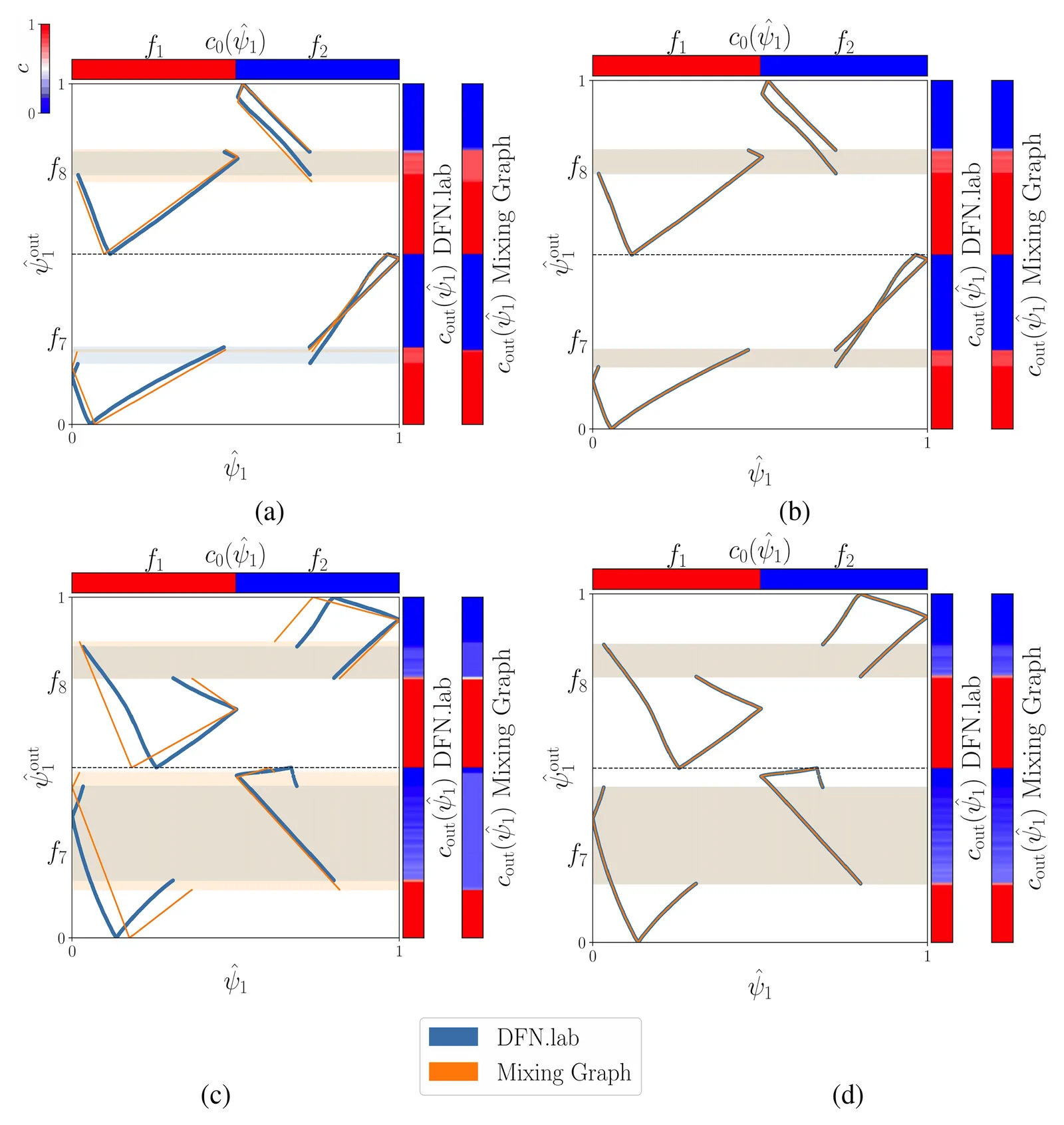
Comparison of the 1D mixing plots for DFN *A* (a,b) and DFN *B* (c,d) generated by DFN.lab and ***𝒢_M_*** under the PWI (a,c) and PWS (b,d) models. Colour bars denote inlet $c_{0}\left({\hat{\psi }}_{1}\right)$ and outlet $c_{\text{out}\;}\left({\hat{\psi }}_{1}\right)$ concentration distributions and shaded bands indicate overlap regions of intersection mixing leading to $0<c_{\text{out}\;}\left({\hat{\psi }}_{1}\right)<1$.

Figure 13 compares the 1D mixing plots (see details in Appendix D) for both test DFNs generated by DFN.lab and ***𝒢_M_*** under both the PWI and PWS models. Figure 13a and Table 1 show that the PWI model fairly accurately captures the mixing dynamics generated by DFN.lab for DFN *A* in terms of both the outlet concentration distribution $c_{\text{out}\;}\left({\hat{\psi }}_{1}\right)$ and mapping ${\hat{\psi }}_{1}\mapsto {\hat{\psi }}_{1}^{\text{out}\;}$, although some minor differences are apparent. In these plots CS of fluid elements is clearly shown by the cut and shuffled 1D lines in the ${\hat{\psi }}_{1}-{\hat{\psi }}_{1}^{\text{out}\;}$ plane, as well as the impact of intersection mixing which dilutes the 1D outlet concentration field as $0<c_{\text{out}\;}\left({\hat{\psi }}_{1}\right)<1$. A direct result of the PWI model is that it produces straight lines in the ${\hat{\psi }}_{1}-{\hat{\psi }}_{1}^{\text{out}\;}$ plane as fluid parcels are purely cut and shuffled in the *ψ*_1_ coordinate without deformation (stretching or compression), hence the mapping ${\hat{\psi }}_{1}\mapsto {\hat{\psi }}_{1}^{\text{out}\;}$ is piecewise linear. Conversely, the DFN.lab results show some evidence of streamline deformation, as indicated by the slight curvature of lines in the ${\hat{\psi }}_{1}-{\hat{\psi }}_{1}^{\text{out}\;}$ plane.

Figure 13b and Table 1 shows that for DFN *A*, the PWS model predicts the outlet concentration $c_{\text{out}}\left({\hat{\psi }}_{1}\right)$ and streamfunctions ${\hat{\psi }}_{1}^{\text{out}\;}$ almost exactly due the resolution of fluid deformation during advective mixing. The difference between the PWI and PWS models is more pronounced for DFN *B*, consistent with the more disordered nature of this DFN. Figure 13c shows a significant level of discrepancy between the PWI model and the DFN.lab results, whereas the PWS model (Figure 13d) accurately reproduces the mixing dynamics generated by DFN.lab. As many fracture networks typically exhibit a much larger level of disorder than DFN *B*, the impact of fluid deformation upon advective mixing is likely to be more pronounced than that indicated in Figure 13(c,d).

**Figure 14:**
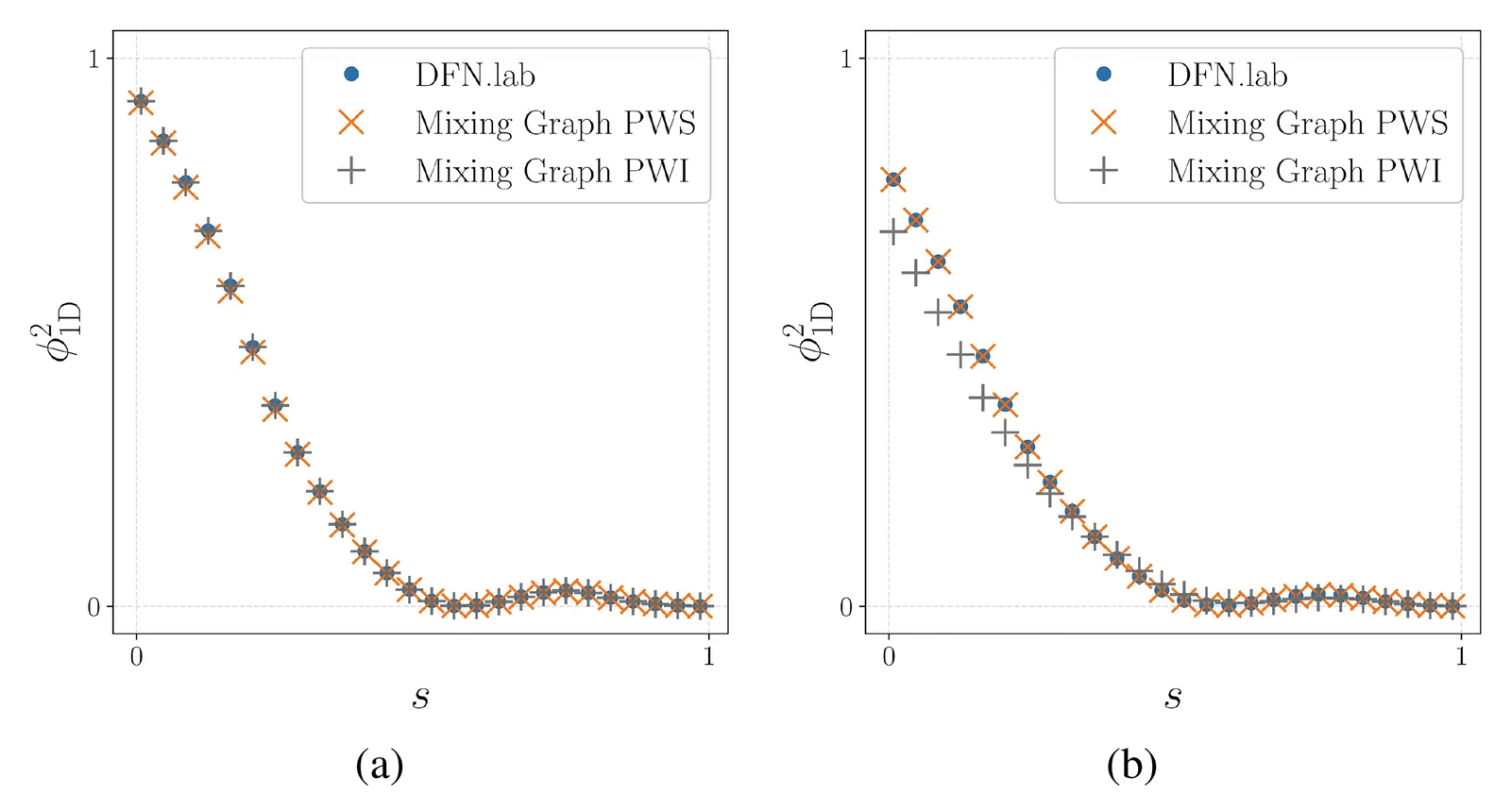
Comparison of multi-scale variance $\phi_{1\text{D}}^{2}(s)$ for (a) DFN *A* and (b) DFN *B* between DFN.lab results (blue dots) and the PWS (orange crosses) and PWI (grey crosses) models for ***𝒢_M_***.

Figure 14 shows that that while the PWS model results very closely match the DFN.lab results for both test DFNs, the results from the PWI model slightly deviates at small to intermediate scales for DFN *A*, and in a more pronounced fashion for DFN *B*. This is consistent with the ${\text{Φ}}_{1\text{D}}^{2}$ values in Table 1, which indicates that the PWI model for DFN *A* and *B* incurs respective mix-norm errors of 1.4% and 10.5%, whereas that the PWS model is highly accurate. The excellent level of agreement between DFN.lab and the PWS model confirms that advective mixing in fracture networks follows a PWS discontinuous mixing process, and indicates that advective mixing can be accurately predicted via the PWS mixing graph.

### 4.4 Advective Mixing in Iterated Fracture Networks

**Figure 15:**
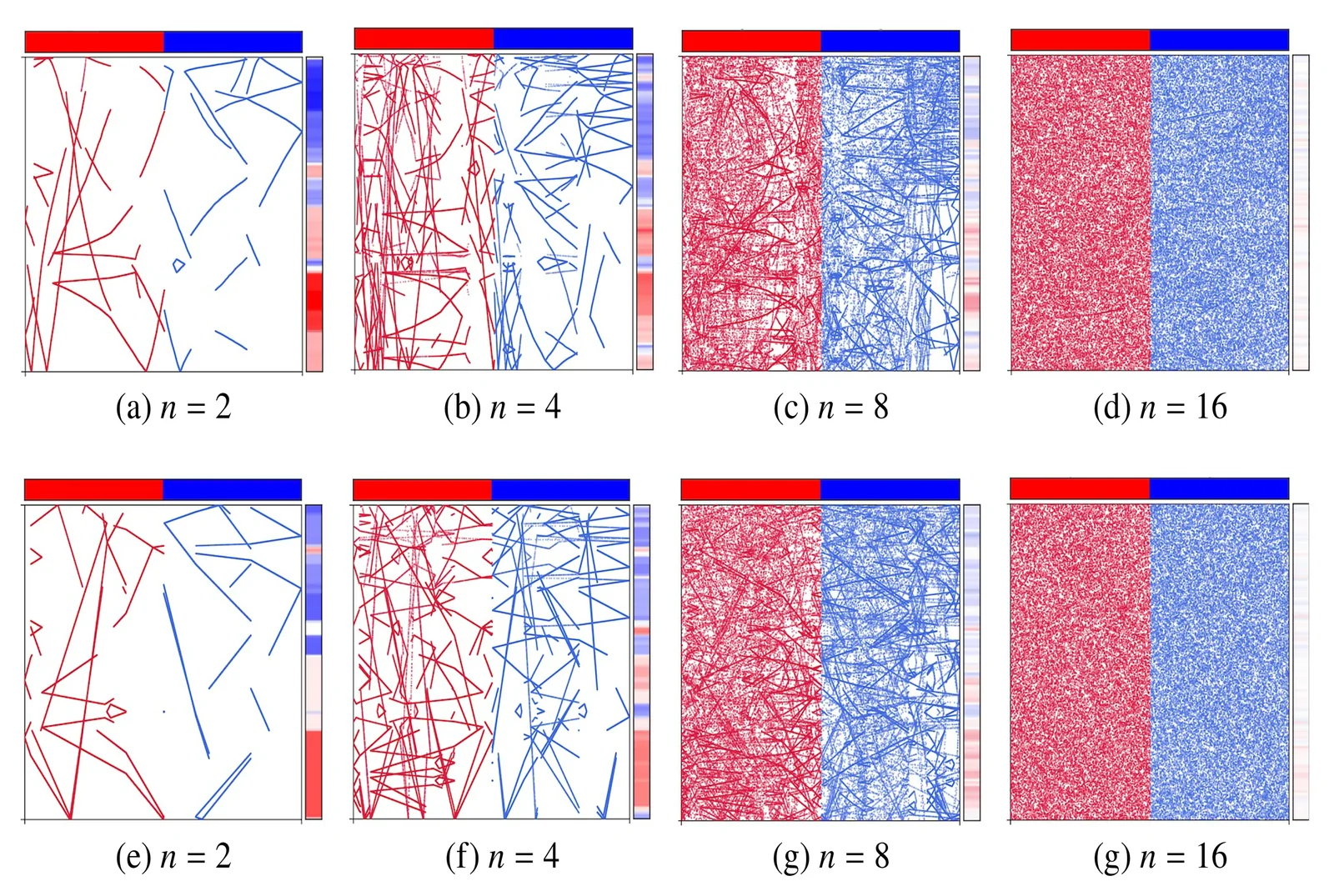
Evolution of 1D mixing plots for iterated mixing in DFN B under the (top row) PWS and (bottom row) PWI mixing graph representations after *n* = 2, *n* = 4, *n* = 8, and *n* = 16 iterations.).

**Figure 16:**
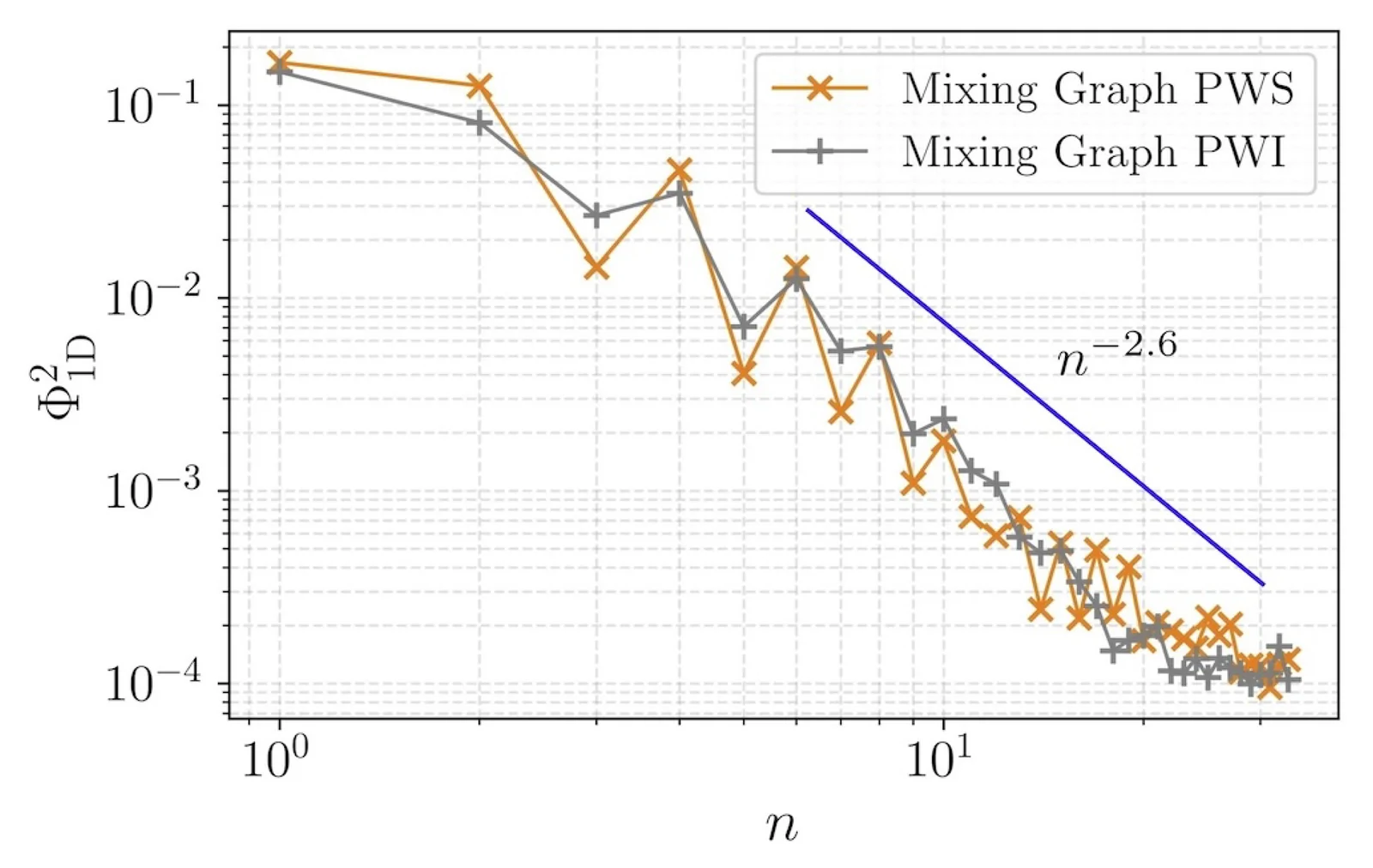
Decay of 1D mix-norm ${\text{Φ}}_{1\text{D}}^{2}(n)$ as a function of iteration number *n* for the orthogonal DFN under the PWS and PWI models.

To highlight the utility of the graph-based approach, we also consider *iterated* mixing in DFN *B*, where the outlet concentration distribution $c_{\text{out}\;}\left({\hat{\psi }}_{1}\right)$ for one iteration of the network forms the inlet concentration distribution $c_{0}\left({\hat{\psi }}_{1}\right)$ for the following iteration. The 1D mixing plots in Figure 15 show that under both the PWI and PWS models, this DFN exhibits rapid and complete mixing with increasing iteration number *n*, where the outlet concentration *c*_out_ → 1/2 as *n* → ∞. Similarly, Figure 16 shows that the 1D mix-norm ${\text{Φ}}_{1\text{D}}^{2}(n)$ for both models converges toward zero with *n* as a power-law $\left({\text{Φ}}_{1\text{D}}^{2}(n)\sim n^{-7}\right)$, reflecting the weak ergodic mixing nature (algebraic decay) of the PWI and PWS transforms. Figure 16 also shows that ${\text{Φ}}_{1\text{D}}^{2}(n)$ is quite similar between the PWI and the PWS models, suggesting that although the PWI model does not resolve the detailed mixing dynamics, this has minimal impact on quantifying mixing in larger fracture networks.

## 5 Conclusions

In this study, we identify the fundamental mechanisms controlling advective mixing in three-dimensional (3D) DFNs and leverage these insights to develop a parsimonious, graph-based representation of advective mixing. We examine the Lagrangian kinematics of 3D DFNs under steady, isotropic Darcy flow. Although this assumption precludes chaotic advection, such effects are likely negligible given the highly constrained geometry of fracture networks. Instead, the intrinsic topological complexity of fracture networks gives rise to stagnation points and critical lines that regulate mixing through streamline routing.

We show that mixing is governed by discontinuous mixing, whereby fluid elements undergo cutting and shuffling (CS) as streamlines are routed through fractures and their intersections. We identify two distinct mechanisms: fracture mixing and intersection mixing, which respectively control mixing within individual fractures and at their intersections. These mechanisms operate over widely separated length scales: fracture mixing cuts and shuffles fluid elements at the fracture scale *L*, whereas intersection mixing acts at the much smaller aperture scale 𝓁, with 𝓁 ≪ *L*. This CS process is illustrated in Figure 11, which shows how the tracer concentration field *c*_0_ over the inlets to the DFN (Figure 1b,c) is mapped to the outlet concentration distribution via cutting and shuffling of fluid elements.

Hence the mixing dynamics in DFNs differ fundamentally from those in granular media, where mixing arises purely through continuous stretching and folding (SF) of fluid elements, and also from 3D porous networks, which exhibit a combination of continuous (SF) and discontinuous cutting and shuffling (CS) dynamics (Lester *et al*. 2025*a*). As the geometry of porous materials becomes increasingly constrained—from granular media to pore networks to fractured networks—the dominant mixing behavior correspondingly transitions from continuous (SF), to mixed (SF/CS), and ultimately to predominantly discontinuous (CS) dynamics.

The streamfunction coordinate system is used to construct intersection ***ℳ_I_*** and fracture ***ℳ_F_*** mixing maps that compactly encode advective mixing. We consider two variants of the intersection map ***ℳ_I_*** : a piecewise smooth (PWS) model that accounts for fluid deformation arising from velocity fluctuations, and a piecewise isometric (PWI) model that neglects this effect. These maps are embedded within a mixing graph ***𝒢_M_*** — an enriched extension of the intersection graph ***𝒢_I_*** (Ahuja *et al*. 1993) — that represents the topology of connected fractures and intersections. The mixing graph ***𝒢_M_*** enables efficient streamline routing through the fracture network and predictive modeling of advective mixing in DFNs. Via CS, the PWI and PWS models both encode mixing interfaces that grow linearly from intersection to intersection in the network, which is consistent with the algebraic growth rates observed by Hallack *et al*. (2025).

The PWS and PWI formulations of the mixing graph ***𝒢_M_*** are evaluated against numerical simulations of flow and transport in two test DFNs using the DFN.lab software package. The PWS model reproduces the mixing dynamics with high accuracy (𝒪(10^−5^)), confirming that the underlying discontinuous mixing is piecewise smooth and characterized by cutting and shuffling coupled with fluctuating fluid stretching. In contrast, the PWI mixing graph representation yields small errors (𝒪(10^−2^)) for one test DFN and larger errors (𝒪(10^−1^)) for a more disordered network, indicating that structural disorder promotes fluid deformation. Despite these discrepancies at the local scale, the PWS and PWI models exhibit quite similar mixing behavior (with algebraic decay of mixing measures) over larger fracture networks. We also note that a modification of the PWI method provides an excellent approximation of the PWS method; although beyond the scope of thus study, this method shall be explored further in future.

This convergence suggests that deformation-induced effects may become negligible at larger scales. Overall, we find that although advective mixing in fracture networks is only weakly ergodic, it remains efficient due to interplay of fine-scale intersection mixing at length scale 𝓁 and fracture-scale mixing at length scale *L* efficiently which brings fluid elements from disparate regions of the network into contact. Together, these complementary mechanisms drive advective mixing in 3D DFNs.

The mixing graph framework readily supports upscaled analytic and numerical models of mixing and transport in large fracture networks, for example through stochastic formulations or random graphs conditioned on laboratory or field observations. The key DFN properties that govern advective mixing are (i) the distribution of fluid fluxes between fractures and their intersections, and (ii) the network topology defined by these connected elements. As the distribution of fluxes at fractures and intersections control the advective mixing process, fracture networks with more uniform flux distributions (given the same topology) generate more efficient mixing and highly non-uniform flux distributions lead to “short-circuiting” involving inefficient CS actions that are not even distributed throughout *ψ*_1_, *ψ*_2_. The quantification of these properties in large-scale fracture networks would then provide a solid foundation for development of predictive theories of advective mixing, as well as efficient numerical methods for simulation of transport and mixing.

The advent of such tools and insights establishes a rigorous framework for future studies of fluid-borne processes in fractured media. As shown in Figures 10 and 11, advective mixing in 3D DFNs is controlled by separating streamsurfaces that encode the series of CS actions that evolve the 2D concentration distribution *c*_*n*_ (*ψ*_1,*n*_, *ψ*_2,*n*_) at each face *f_n_* in the network. While the mixing maps ***ℳ_I_***, ***ℳ_F_*** can be used to advect arbitrary streamlines, such that the streamfunctions are mapped from face to face throughout the network as $\left(\psi_{1,n},\psi_{2,n}\right)\overset{{\text{ℳ}}_{I},{\text{ℳ}}_{F}}{\mapsto }\left(\psi_{1,m},\psi_{2,m}\right)$, they can also be employed to resolve these separating streamsurfaces and construct advective maps ***𝒜_F_***, ***𝒜_F_*** that propagate the local concentration field *c*_*n*_ throughout the network as $c_{n}\overset{A_{I},A_{F}}{\mapsto }c_{m}$. This framework not only provides an efficient means of propagating the concentration field under advective mixing, but also establishes a foundation for modeling time-dependent processes such as solute mixing and dispersion, chemical reactions and biological activity.

For example, given the residence-time distribution *τ_n_* over each fracture *n* in the DFN (assuming negligible travel time *τ_n_* = 0 over an intersection), then the distribution of Lagrangian travel times $t_{n}\left(\psi_{1,n},\psi_{2,n}\right)=\sum_{i=1}^{n}\tau_{i}$ over each face generates the temporal maps ***𝒯_F_***, ***𝒯_I_*** = ***I*** that propagate *t*_*n*_ through the network. For solute mixing, methods that combine CS and diffusion processes (Ashwin *et al*. 2002; Kreczak 2019) can be used to generate diffusive maps ***𝒟_F_*** = ***𝒟_F_*** (***𝒜_F_***, ***𝒯_F_***), ***𝒟_I_*** = ***𝒜_I_*** that propagate the solute concentration *c*_*n*_ throughout the network. This allows solute mixing in intersections to be properly resolved for finite transverse Péclet numbers (*Pe*_⊥_ in (3.24)), without resorting to extreme approximations such as complete mixing or streamline routing at intersections. Conversely, solute mixing in fractures is governed by the longitudinal Péclet number (*Pe*_| |_) where *Pe*_||_= *L*/𝓁 *Pe*_⊥_. Similar approaches can also be developed for longitudinal and transverse dispersion and reactive transport.

## Supplementary Material

Appendix

## Figures and Tables

**Table 1:** Comparison of 1D mix-norm values ${\text{Φ}}_{1\text{D}}^{2}$ for the test DFNs between the DFN.lab results and the PWI and PWS models for ***𝒢_M_***.

DFN	Model	${\text{Φ}}_{\text{1D,DNS}}^{2}$	${\text{Φ}}_{1\text{D},{\text{𝒢}}_{\text{M}}}^{2}$	Relative error
*A*	PWI	0.207874	0.204952	1.4 × 10^–2^
*A*	PWS	0.207874	0.207885	5.1 × 10^–5^
*B*	PWI	0.171267	0.153298	1.05 × 10^–1^
*B*	PWS	0.171267	0.171252	8.6 × 10^–5^

## Data Availability

The numerical codes and relevant data will be made available upon publication

## Footnotes

† In the dynamical systems literature fluid stirring is often referred to simply as mixing (Arnold & Avez 1968), whereas in the physical sciences mixing typically denotes the combined action of advection and diffusion (Villermaux 2019). Here we distinguish *advective mixing*, arising purely from fluid deformation, from *solute mixing*, which results from the combined effects of advection and diffusion.
