# Supplementary material for: Cutting and Shuffling Control Mixing in Three-Dimensional Discrete Fracture Networks
Source: J Fluid Mech. Author manuscript; Available in PMC 2026 Nov 2. (PMC7619689; doi:10.1017/jfm.2026.11623)
Supplement: Supplementary file 1 [file EMS213763-supplement-Appendix.pdf]

Appendix A. Chaotic Advection in Fracture Networks

A.1. *Estimation of Lyapunov Exponent in Fracture Networks*

Lester *et al.* (2025*a*) show that the Lyapunov exponent $\hat{\lambda}_\infty$ in ordered pore networks is controlled by the orientation angles δ, Δ between manifolds as

$$\hat{\lambda}_\infty(\delta,\Delta) = \max(\ln(|\xi| \pm \sqrt{\xi^2-1}), 0), \quad \xi = \frac{9}{8}\cos\delta - \frac{1}{8}\cos(2\Delta+\delta), \tag{A 1}$$

with $\delta \in [-\pi,\pi]$, $\Delta \in [-\pi,\pi]$. For random pore networks with δ, Δ both uniformly distributed between $-\pi$ and π, the average Lyapunov exponent is given by the ensemble average

$$\lambda_\infty \equiv \frac{1}{4\pi^2}\int_{-\pi}^{\pi}\int_{-\pi}^{\pi}\hat{\lambda}_\infty(\delta,\Delta)\,d\delta\,d\Delta \approx 0.1178. \tag{A 2}$$

For fracture networks, these angles are constrained as $\delta \in [-\alpha,\alpha]$, $\Delta \in [-\alpha,\alpha]$, where $\alpha = \arctan(\ell/L) \approx \ell/L \ll 1$. Assuming these angles are uniformly distributed in fracture networks within these limits, then $\hat{\lambda}_\infty$ is symmetric about $\delta = 0$, $\Delta = 0$,

$$\lambda_\infty \equiv \frac{1}{2\alpha^2}\int_{0}^{\alpha}\int_{-\alpha}^{\alpha}\hat{\lambda}_\infty(\delta,\Delta)\,d\delta\,d\Delta. \tag{A 3}$$

As $\hat{\lambda}_\infty > 0$ for $\Delta > 0$ and $-\Delta/2 < \delta < \Delta$, then to leading order the δ integral in (A 3) can be approximated by Simpson's rule as

$$\int_{-\alpha}^{\alpha}\hat{\lambda}_\infty(\delta,\Delta)\,d\alpha = \int_{-\Delta/2}^{\Delta}\hat{\lambda}_\infty(\delta,\Delta)\,d\alpha \approx \frac{3\Delta}{4}\frac{4}{3}\hat{\lambda}_\infty(\delta=\Delta/4,\Delta) \approx \frac{3}{4}\Delta^2 + O(\Delta^3). \tag{A 4}$$

Integrating with respect Δ over $\Delta \in [0,\alpha]$ then yields

$$\lambda_\infty \approx \frac{1}{2\alpha^2}\frac{\alpha^3}{4} = \frac{\alpha}{8} \approx \frac{1}{8}\frac{\ell}{L} \ll 1. \tag{A 5}$$

A.2. *Localisation of Helicity Density in Fracture Networks*

We can show that under Stokes flow the helicity density is localised to fracture boundaries by consideration of the helicity density evolution equation (Moffatt 1969) that arises from the

incompressible Navier-Stokes and vorticity equations

$$\frac{D\mathbf{v}}{Dt} = -\frac{1}{\rho}\nabla p + \nu\nabla^2\mathbf{v}, \quad \nabla\cdot\mathbf{v} = 0, \tag{A 6}$$

$$\frac{D\boldsymbol{\omega}}{Dt} = \boldsymbol{\omega}\cdot\nabla\mathbf{v} + \nu\nabla^2\boldsymbol{\omega}, \quad \nabla\cdot\boldsymbol{\omega} = 0, \tag{A 7}$$

as

$$\frac{Dh}{Dt} = \frac{D\mathbf{v}}{Dt}\cdot\boldsymbol{\omega} + \frac{D\boldsymbol{\omega}}{Dt}\cdot\mathbf{v} = \nu\left[\mathbf{v}\cdot\nabla^2\boldsymbol{\omega} + \boldsymbol{\omega}\cdot\nabla^2\mathbf{v}\right] + \nabla\cdot\left[\boldsymbol{\omega}\left(-\frac{p}{\rho} + \frac{1}{2}v^2\right)\right], \tag{A 8}$$

where the first term on the RHS represents viscous helicity dissipation, and the second term on the RHS represents conservative helicity density flux. Hence helicity density is dissipated in the bulk and is only generated at boundaries (Moffatt 1969).

Appendix B. Quantification of Mixing Maps $\mathcal{M}_I$, $\mathcal{M}_F$

B.1. *Quantification of the Intersection Mixing Map $\mathcal{M}_I$*

B.1.1. *Differentiation of PWI and PWS transforms*

The difference between the PWI and PWS transforms can be clearly demonstrated for the case of flow through a T-shaped intersection such as the $\hat{\psi}_1$-streamsurface shown in Figure 4e. Under the assumption of zero flow along the intersection, streamlines are confined to $\hat{\psi}_1$-streamsurfaces that are also planes of constant x_1. Denoting the faces in Figure 4e as $f_1 \equiv f_1^i$, $f_2 \equiv f_1^o$, $f_3 \equiv f_2^o$, an overall flux balance gives $Q_1 = Q_2 + Q_3$. An areal flux balance within a $\hat{\psi}_1$-streamsurface also means that the areal fluxes $q_n(x_1)$ (3.7) are related as

$$q_1(x_1) = \int_0^1 ||\mathbf{v}_1||d\psi_{2,1} = Q_1\nabla\psi_{1,1}, \tag{B 1}$$

$$q_2(x_1) = \int_0^1 ||\mathbf{v}_2||d\psi_{2,2} = Q_2\nabla\psi_{1,2} = \int_0^{\psi_{2,1}^\star} ||\mathbf{v}_1||d\psi_{2,1} = \psi_{2,1}^\star Q_1\nabla\psi_{1,1} = \psi_{2,1}^\star q_1(x_1), \tag{B 2}$$

$$q_3(x_1) = \int_0^1 ||\mathbf{v}_3||d\psi_{2,3} = Q_3\nabla\psi_{1,3} = \int_{\psi_{2,1}^\star}^1 ||\mathbf{v}_1||d\psi_{2,1} = (1-\psi_{2,1}^\star)Q_1\nabla\psi_{1,1} = (1-\psi_{2,1}^\star)q_1(x_1), \tag{B 3}$$

where $\psi_{2,1}^\star$ is the $\psi_{2,1}$ value of the separating streamline in the inlet. Extending this relationship along the entire longitudinal coordinate x_1 of the intersection, $\psi_{2,1}^\star(x_1)$ varies with x_1 as

$$\psi_{2,1}^\star(x_1) = \frac{q_2(x_1)}{q_1(x_1)} = 1 - \frac{q_3(x_1)}{q_1(x_1)}, \tag{B 4}$$

hence is $\psi_{2,1}^\star(x_1)$ invariant along the intersection if the ratio of areal fluxes is constant throughout, i.e.

As only critical features such as separating streamlines control streamline routing and advective mixing, the condition $q_1(x_1)/q_2(x_1) = \text{const.}$ means that intersection mixing does not vary with x_1, and the separating streamline only depends upon the volumetric fluxes as

$$\psi_{2,1}^\star = \frac{Q_2}{Q_1} = 1 - \frac{Q_3}{Q_1}. \tag{B 5}$$

Thus intersection mixing may be determined completely from the volumetric fluxes Q_1, Q_2, Q_3. This formulation (B 5) is consistent with the PWI transform, where stretching due to velocity fluctuations

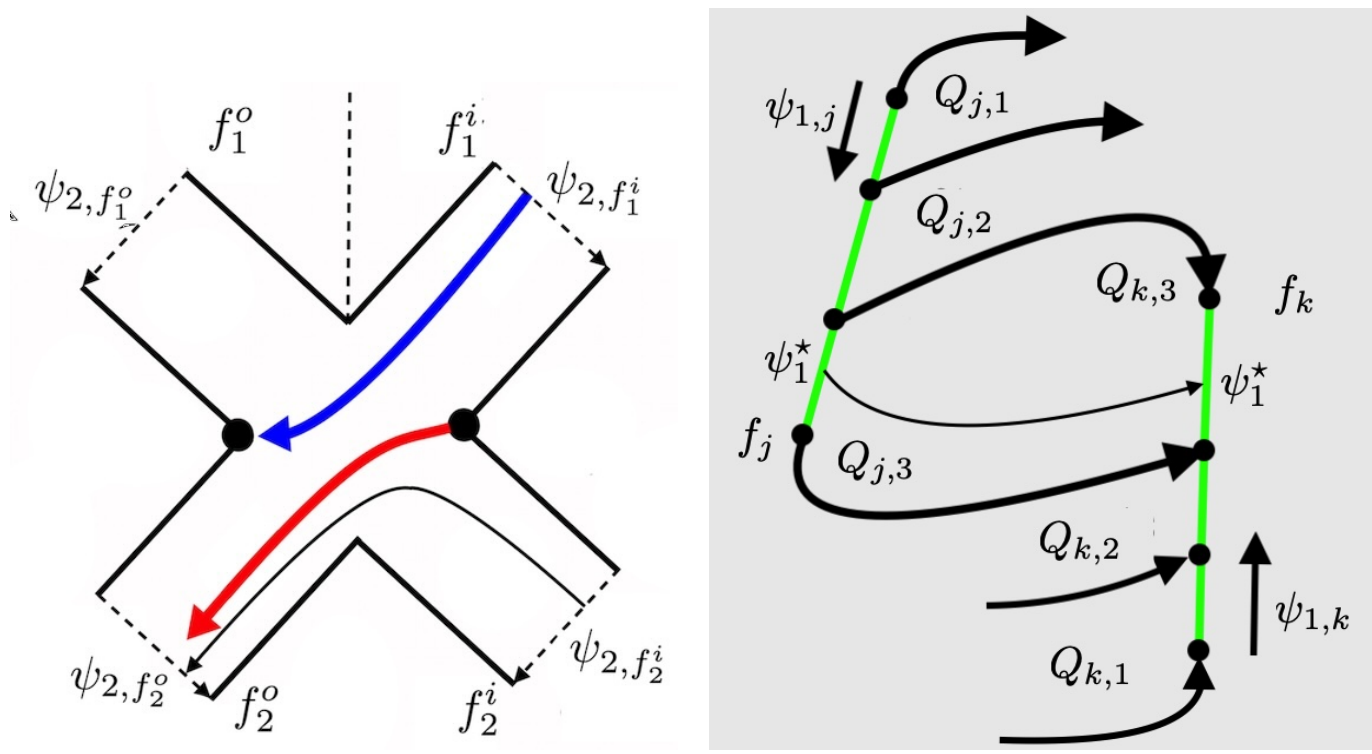

(a) (b)

Figure 17: (a) Schematic supporting flux balance (B 8) for the streamline topology shown in Figure 4b. The dotted vertical line depicts an arbitrary reference line corresponding to $Q_\psi = 0$, and the local $\psi_{2,n}$ streamfunctions at each face f_n are shown. The black line depicts a typical streamline routed through the intersection. (b) Schematic of supporting flux balances (B 19), (B 21) for fracture mixing based on typical streamline topology (such as shown in Figure 1c.) around a pair of intersections (green lines). Separating streamlines (thick black lines) connect to critical points (black dots), routing topologically distinct"parcels" of streamlines with the fracture. The critical streamnumber $\psi_1^\star$ identifies a given streamline (thin black line) connecting face f_j to f_k.

are negligible, whereas the variable flux case (B 4) is consistent with the PWS transform. As the local $\psi_{1,n}$ streamfunction also depends upon the areal fluxes, if $q_1(x_1)/q_2(x_1) = \text{const.}$, then these are equivalent as

$$\psi_{1,1}(x_1) = \frac{1}{Q_1}\int_0^{x_1} q_1(x_1')dx_1' = \frac{1}{Q_2}\int_0^{x_1} q_2(x_1')dx_1' = \psi_{1,2}(x_1), \tag{B 6}$$

hence all the $\psi_{1,n}$ streamfunctions are invariant over an intersection under the PWI model.

These concepts can be readily generalised to the various intersection and streamline topologies shown in Figure 4, leading to the result the intersection mixing dynamics along x_1 are also invariant if the ratio of areal fluxes $q_j(x_1)/q_k(x_1) = \text{const.}$ for all faces f_j, f_k adjacent to a common intersection. Under this condition, the local ψ_1-streamfunctions are invariant, i.e. $\psi_{1,j} = \psi_{1,k}$ for all faces f_j, f_k, and intersection mixing is completely characterised via the PWI transform in terms of the volumetric fluxes Q_j, Q_k, etc. Conversely, if any of the areal flux ratios $q_j(x_1)/q_k(x_1) \neq \text{const.}$, then $\psi_{1,j} \neq \psi_{1,k}$ and so local streamfunctions are not invariant and the PWS transform is required to resolve the variable mixing dynamics along the intersection.

To explicitly quantify the intersection mixing map $\mathcal{M}_I$ for the PWI (3.8) and PWS (3.9) transforms, we now consider the routing of streamlines through a $\hat{\psi}_1$-streamsurface embedded in intersection as they flow from an inlet face $f_{n^i}^i$ to an outlet face $f_{n^o}^o$ as shown in Figure 3b. This routing is governed by the local streamfunctions $(\psi_{1,n}, \psi_{2,n})$ defined on each face f_n.

B.1.2. *Piecewise Isometric (PWI) Model*

As detailed above, the local $\psi_{1,n}$ streamfunction of face f_n is invariant with respect to x_1 under the PWI model. For a given intersection, this streamfunction is invariant across all inlet f_n^i and outlet f_n^o faces, i.e.

$$\psi_{1,f_1^i}(x_1) = \dots \psi_{1,f_{N^i}^i}(x_1) = \psi_{1,f_1^o}(x_1) = \dots \psi_{1,f_{N^o}^o}(x_1). \tag{B 7}$$

In this case streamline routing depends only on the distribution of flow rates Q_n through the faces of I_m and the inlet streamline face number and ψ_2 streamnumber. Under the PWI model, the intersection

map $\mathcal{M}_I$ returns the outlet face f_{n^o} and local ψ_2 streamfunction ψ_{2,n^o} given the inlet face f_{n^i} and local ψ_2 streamfunction ψ_{2,n_i}. As shown in Figure 17a, this map can be quantified by considering a flux balance over the inlets and outlets of the intersection, where the inlet and outlet faces and local ψ_2-streamfunctions are connected via a common streamline. By choosing an arbitrary reference line (dotted vertical line in Figure 17a), the inlet (outlet) faces of the intersection can be numbered in ascending order from this line in the clockwise (counter-clockwise) direction as f_n^i, with $n = 1 : N_i$ (f_n^o, $n = 1 : N_o$), where N_i (N_o) is the total number of inlets (outlets) of the intersection. Similarly, the local ψ_2 streamfunctions for the inlets (outlets) also increase in the same clockwise (counter-clockwise) direction.

As the flux Q_n^i (Q_n^o) through inlet (outlet) face f_n^i (f_n^o) between streamlines $\psi_{2,n} = 0$ and $\psi_{2,n} = \psi$ is $\psi\, Q_n^i$ ($\psi\, Q_n^o$), then the cumulative flux Q_{ψ_2} into and out of the intersection from the reference line to a given streamline through face f_{n^i} with inlet ψ_2 streamfunction ψ_{2,n^i} is then

$$\sum_{n=1}^{n^i-1} Q_n^n + Q_{n^i}^i\, \psi_{2,f_{n^i}^i} = \sum_{n=1}^{n^o-1} Q_n^o + Q_{n^o}^o\, \psi_{2,f_{n^o}^o} \equiv Q_{\psi_2}. \tag{B 8}$$

Note that this equation holds for any intersection and 2D streamline topology, regardless of the number and arrangement of inlets and outlets. From this cumulative flux, the outlet face index n^o is computed as

$$\sum_{n=1}^{n^o-1} Q_n^o \leqslant Q_{\psi_2} < \sum_{n=1}^{n^o} Q_n^o, \tag{B 9}$$

and by re-arranging (B 8), the local streamfunctions on the outlet face n^o are given as

$$\psi_{1,f_{n^o}^o} = \psi_{1,f_{n^i}^i} \qquad \psi_{2,f_{n^o}^o} = \frac{Q_{\psi_2} - \sum_{j=1}^{n^o-1} Q_n^o}{Q_{n^o}^o}. \tag{B 10}$$

Equations (B 9), (B 10) define the map $\mathcal{M}_I$ which is piecewise isometric in that streamlines are cut and shuffled with respect to the local ψ_2 coordinate, while ψ_1 remains unchanged.

B.1.3. *Piecewise Smooth (PWS) Model*

For the piecewise smooth (PWS) model, the PWI assumption (B 7) is relaxed, such that in general the local ψ_1 streamfunctions at each face f_n of the intersection may have different values at the same x_1 coordinate along the intersection. The mapping between the $\psi_{1,n}$ and x_1 coordinates for each face of the intersection is captured by the monotone maps

$$\Psi_{1,n}(x_1), \qquad X_{1,n}(\psi_1), \tag{B 11}$$

where $X_{1,n}$ is the inverse of $\Psi_{1,n}$ and $\Psi_{1,n}$ can be determined from the areal flux $q_n(x_1)$ through face f_n as

$$\Psi_{1,n}(x_1) \equiv \psi_{1,n}(x_1) = \frac{1}{Q_n}\int_0^{x_1} q_n(x_1')dx_1', \tag{B 12}$$

which is monotone increasing as $\mathbf{v}_n(\mathbf{x})$ is positive, hence the inverse function $X_{1,n}(\psi_1)$ is well defined. As the streamlines entering and leaving the intersection have the same x_1 coordinate, the PWS analogue of (B 13) is

$$x_1 = X_{1,f_1^i}(\psi_{1,f_1^i}) = \cdots = X_{1,f_{N^i}^i}(\psi_{1,f_{N^i}^i}) = X_{1,f_1^o}(\psi_{1,f_1^o}) = \cdots = X_{1,f_{N^o}^o}(\psi_{1,f_{N^o}^o}). \tag{B 13}$$

Streamline routing then can be determined by first computing the x_1 coordinate for an incoming streamline as

$$x_1 = X_{1,n^i}\left(\psi_{1,n^i}\right), \tag{B 14}$$

where, using the same notation as for the PWI model, n_i denotes the inlet face that contains the streamline. Following the same approach as for the PWI model, we define the cumulative areal flux as

$$\sum_{n=1}^{n^i-1} q_n^i(x_1) + q_{n^i}^i(x_1)\,\psi_{2,f_{n^i}^i} = \sum_{n=1}^{n^o-1} q_n^o(x_1) + q_{n^o}^o(x_1)\,\psi_{2,f_{n^o}^o} \equiv q_{\psi_2}. \tag{B 15}$$

From this cumulative flux, we can compute the outlet face index n^o via

$$\sum_{n=1}^{n^o-1} q_n^o(x_1) \leqslant q_\psi < \sum_{n=1}^{n^o} q_n^o(x_1), \tag{B 16}$$

and by re-arranging (B 15), the local streamfunctions on the outlet face are given as

$$\psi_{1,f_{n^o}^o} = \Psi_{1,f_{n^o}^o}(x_1) \qquad\qquad \psi_{2,f_{n^o}^o} = \frac{q_\psi - \sum_{n=1}^{n^o-1} q_n^o(x_1)}{q_{n^o}^o(x_1)}. \tag{B 17}$$

Equations (B 16), (B 17) define the map $\mathcal{M}_I$ under the PWS model.

B.2. *Quantification of the Fracture Mixing Map* $\mathcal{M}_F$

As shown in Figures 1c and 5, fracture mixing arises from streamline routing within fractures between intersections. As the local ψ_2 streamfunction is considered to be invariant over a fracture, fracture mixing is quantified solely in terms of the local ψ_1 streamfunctions flowing from outlet face f_j ($\psi_{1,j}$) and to inlet face f_k ($\psi_{1,k}$). Figure 17b shows that separating streamlines connected to critical points divide the total flux Q_j from or into face f_j into coherent streamline "parcels" with fluxes $Q_{j,n}$ with $n = 1 : N_j$ such that $Q_j = \sum_{n=1}^{N_j} Q_{j,n}$. These parcels are ordered in the same manner as the local ψ_1 streamfunction, which is ordered such that for outlet (inlet) faces it is increasing in a clockwise (counter-clockwise) around a 1D intersection line. Mapping of $\psi_{1,j}$ to $\psi_{1,k}$ via the fracture mixing map $\mathcal{M}_F$ is then given in terms of the flow rates leaving f_j and entering f_k as

$$Q_j\psi_{1,j} = \sum_{n=1}^{n^o-1} Q_{j,n} + \psi_1^\star Q_{j,n^o}, \tag{B 18}$$

where $Q_j = \sum_{n=1}^{N_j} Q_{j,n}$ is the total flow rate flowing through outlet face f_j, where the critical streamnumber $\psi_1^\star$ quantifies the proportion of the flux Q_{j,n^o} that corresponds to the streamline quantified by $\psi_{1,j}$. In (B 18), n_o is the number of the streamline parcel that contains the streamline with local streamfunction $\psi_{1,j}$, which satisfies

$$\sum_{n=1}^{n^o-1} Q_{j,n} < Q_j\psi_{1,j} \leqslant \sum_{n=1}^{n^o} Q_{j,n}. \tag{B 19}$$

From (B 18) and (B 19), both n^o and ψ_1 can be computed directly from the flow rates $Q_{j,n}$ leaving f_j and the streamfunction $\psi_{1,j}$. Similarly, $\psi_{1,k}$ and the parcel n_i that contains the streamline entering f_k are computed as

$$Q_k\psi_{1,k} = \sum_{n=1}^{n^i-1} Q_{k,n} + \psi_1^\star Q_{k,n^i}, \tag{B 20}$$

where n_i satisfies

$$\sum_{n=1}^{n^i-1} Q_{k,n} < Q_k\psi_{1,k} \leqslant \sum_{n=1}^{n^i} Q_{k,n}. \tag{B 21}$$

Hence equations (B 18)-(B 21) define the fracture mixing map $\mathcal{M}_F$, and facilitate updating of $\psi_{1,j} \mapsto \psi_{1,k}$ as well as the edge mapping $j \mapsto k$. Together, the mixing maps $\mathcal{M}_I$, $\mathcal{M}_F$ determine the local stream numbers $(\psi_{1,n}, \psi_{2,n})$ and the faces f_n that streamlines visit as they propagate through the network.

The mixing graph $\mathcal{G}_M$ (which quantifies the fracture network topology and fluxes as a WADG), along with the ordering and orientation of fluid parcels from face to face (edge to edge) through out the fracture network (graph) is sufficient to fully characterise the PWI intersection maps $\mathcal{M}_I$ and the fracture maps $\mathcal{M}_F$. For the PWS intersection maps $\mathcal{M}_I$, the monotone maps $\Psi_{1,n}(x_1)$ or $X_{1,n}(\psi_1)$ must also be determined at each face throughout the network. As this can be achieved efficiently via spline functions, both the PWI and PWS models generate parsimonious representations of the Lagrangian kinematics and thus advective mixing within fracture networks.

Appendix C. Mixing Measures

The evolution of the concentration field within the fracture network can be characterised in terms of the *local* concentration mean $\bar{c}_n$ and variance σ_n^2 at each face f_n, which are defined as

$$\bar{c}_n \equiv \int_0^1 \int_0^1 c_n(\psi_1, \psi_2)\, d\psi_1\, d\psi_2 \qquad \sigma_{c_n}^2 \equiv \int_0^1 \int_0^1 [c_n(\psi_1, \psi_2) - \bar{c}_n]^2\, d\psi_1\, d\psi_2. \tag{C 1}$$

Although these measures can vary at each face f_n within the DFN, the global *inlet* concentration mean and variance

$$\bar{c}_0 \equiv \frac{1}{Q} \sum_{n=1}^{N_{I_i}} Q_n \bar{c}_n \qquad \sigma_0^2 \equiv \frac{1}{Q} \sum_{n=1}^{N_{I_i}} Q_n \left(\sigma_{c_n}^2 + \bar{c}_0^2 - \bar{c}_n^2 \right), \tag{C 2}$$

are preserved throughout the fracture network in the sense that the global *outlet* mean and variance are equivalent

$$\bar{c}_{\text{out}} \equiv \frac{1}{Q} \sum_{n=N_I - N_{I_o}}^{N_I} Q_n \bar{c}_n = \bar{c}_0 \qquad \sigma_{\text{out}}^2 \equiv \frac{1}{Q} \sum_{n=N_I - N_{I_o}}^{N_I} Q_n \left(\sigma_{c_n}^2 + \bar{c}_{\text{out}}^2 - \bar{c}_n^2 \right) = \sigma_0^2, \tag{C 3}$$

due to the non-dissipative nature of purely advective mixing. A different mixing measure is the *mix-norm* metric (Mathew *et al.* 2005), a multi-scale mixing measure which robustly measures the degree of mixing by integrating over a scale-dependent concentration variance. The mix-norm is maximal if the concentration field is separated at the largest possible scale (such as the concentration distributions shown in Figure 2(a,b)), and vanishes if and only if the scalar field is perfectly homogenised at the smallest scale (Thiffeault 2012). The mix-norm can be computed from the global outlet concentration field $c_{\text{out}}(\hat{\psi}_1, \hat{\psi}_2)$ in terms of the rescaled (dimensional) global streamfunctions $\hat{\psi}_1' \equiv L\,\hat{\psi}_1 \in [0, L]$, $\hat{\psi}_2' \equiv \ell\,\hat{\psi}_2 \in [0, \ell]$ as follows. First, we define the average of $c_{\text{out}} - \bar{c}_0$ over a square of dimension s centred over $\hat{\psi}_1, \hat{\psi}_2$ as

$$d(s, \hat{\psi}_1', \hat{\psi}_2') \equiv \frac{\ell\, L}{s^2} \int_{\max(\hat{\psi}_1' - s/2, 0)}^{\min(\hat{\psi}_1' + s/2, L)} \int_{\max(\hat{\psi}_2' - s/2, 0)}^{\min(\hat{\psi}_2' + s/2, \ell)} c_{\text{out}}(\xi_1/L, \xi_2/\ell) - \bar{c}_0\, d\xi_2\, d\xi_1. \tag{C 4}$$

Note that for $s > \ell$, all variance in c_{out} with respect to $\hat{\psi}_2'$ is integrated out in (C 4), hence d^2 is not a function of $\hat{\psi}_2'$ for $s > \ell$. The square of this fluctuation is then integrated over $\hat{\psi}_1'$, $\hat{\psi}_2'$ to give the scale-dependent variance $\phi^2(s)$ with $s \in [0, L]$ as

$$\phi^2(s) \equiv \frac{1}{\ell\, L} \int_0^L \int_0^\ell d^2(s, \hat{\psi}_1', \hat{\psi}_2')\, d\hat{\psi}_1'\, d\hat{\psi}_2', \tag{C 5}$$

such that for $s > \ell$, $\phi^2(s)$ only depends on variations in the $\hat{\psi}_1$ direction. Integrating this scale-dependent variance over s yields the 2D mix-norm Φ^2_{2D}

$$\Phi^2_{2D} \equiv \frac{1}{L}\int_0^L \phi^2(c_{out}, s)\, ds = \frac{1}{L}\int_0^{\ell} \phi^2(s)\, ds + \frac{1}{L}\int_{\ell}^{L} \phi^2(s)\, ds, \\ = \Phi^2_{2D,\ell} + \Phi^2_{2D,L} \tag{C 6}$$

which quantifies the degree of mixing from the smallest scale $s = 0$ to the largest scale $s = L$. As shown in (C 6), the mix-norm can be separated into the ($\hat{\psi}_1$,$\hat{\psi}_2$)-dependent *microscopic* mix-norm $\Phi^2_{2D,\ell}$ which quantifies mixing over scales $s \in [0, \ell]$, and the $\hat{\psi}_1$-dependent *macroscopic* mix-norm $\Phi^2_{2D,L}$ which quantifies quantifies mixing over scales $s \in [\ell, L]$. As $\ell \ll L$, then $\Phi^2_{2D,L} \gg \Phi^2_{2D,\ell}$, hence the mix-norm is accurately approximated by the macroscopic mix-norm as

$$\Phi^2_{2D}(c_{out}) = \Phi^2_{2D,L}(c_{out}) + O(\ell/L). \tag{C 7}$$

As the macroscopic mix-norm only depends upon $\hat{\psi}_1$, (C 7) suggests that mixing in fracture networks can be accurately represented as a 1D process.

Appendix D. 1D Mixing Plots

Associated with the 1D mixing representation (3.22) are the *1D mixing plots* shown in Figure 18 which provide a simplified representation of mixing over the entire fracture network. These plots show how the global streamfunction $\hat{\psi}_1$ over the network inlets is mapped to the outlet global streamfunction $\hat{\psi}_1^{out}$ over the outlets, and so represent cutting and shuffling of streamlines with respect to the $\hat{\psi}_1$ coordinate. These plots also show how the step-wise inlet concentration field $c_0(\hat{\psi}_1) = H(1/2 - \hat{\psi}_1)$ (where H is the Heaviside step function) is mapped to the outlet concentration field $c_{out}(\hat{\psi}_1)$, where the colour bars in Figure 18 may be considered as 1D analogues ($\hat{\psi}_1$ only) of the 2D inlet and outlet concentration fields shown in Figure 10.

To clearly explain these plots, we first consider the 1D mixing plot shown in Figure 18a for the trivial case of a fracture network with no mixing ($\hat{\psi}_1^{out} = \hat{\psi}_1$). For this plot, each streamline advected through the system arises as a discrete point (with colour corresponding to $c_0(\hat{\psi}_1)$) in the $\hat{\psi}_1 - \hat{\psi}_1^{out}$ plane. Hence these points form a 45° line which is red from $(0, 0)$ to $(1/2, 1/2)$ and blue from $(1/2, 1/2)$ to $(1, 1)$ and the inlet and outlet concentration distributions are identical as $c(\hat{\psi}_1) = c(\hat{\psi}_1^{out}) = c_{out}(\hat{\psi}_1)$.

Figure 18b shows the 1D mixing plot generated by the network shown in Figure 2a that exhibits fracture mixing only. For this network, streamline routing within fractures generates CS of streamlines with respect to the ψ_1 coordinate, leading to the 2D and 1D concentration distributions shown respectively in Figures 2b and 18b. The latter figure highlights how fracture mixing acts to cut and shuffle sets of points along vertical $\hat{\psi}_1^{out}$ axis, which repositions (and in some cases, reflects) segments of the 45° line shown in Figure 18b. This results in large-scale fracture mixing of the 1D concentration distribution $c(\hat{\psi}_1^{out})$ shown in Figure 18b, which is equivalent to the 2D concentration distribution $c(\psi_{1,n}, \psi_{2,n})$ shown in Figure 2b. Note that although values of the 1D concentration field are unchanged $c_{out}(\hat{\psi}_1) \in \{0, 1\}$, the 1D scale-dependent variance $\phi^2_{1D}(s)$ and mix-norm Φ^2_{1D} decrease due to large-scale cutting and shuffling of the concentration field $c_{out}(\hat{\psi}_1)$. Repeat random fracture mixing processes would cut and shuffle this 1D concentration distribution into smaller contiguous segments, driving $\Phi^2_{1D} \to 0$, but the concentration values would still be limited to $c_{out}(\hat{\psi}_1) \in \{0, 1\}$.

Figure 18c shows the 1D mixing plot generated by the network shown in Figure 2c that exhibits intersection mixing only. For this network, streamline routing within intersections generates CS actions with respect to the ψ_2 coordinate, leading to the 2D and 1D concentration distributions shown respectively in Figures 2d and 18c. For intersection mixing, the points (streamlines) that comprise the 45° line in Figure 18a are cut and shuffled according to their $\hat{\psi}_2$ coordinate. In Figure 18c this

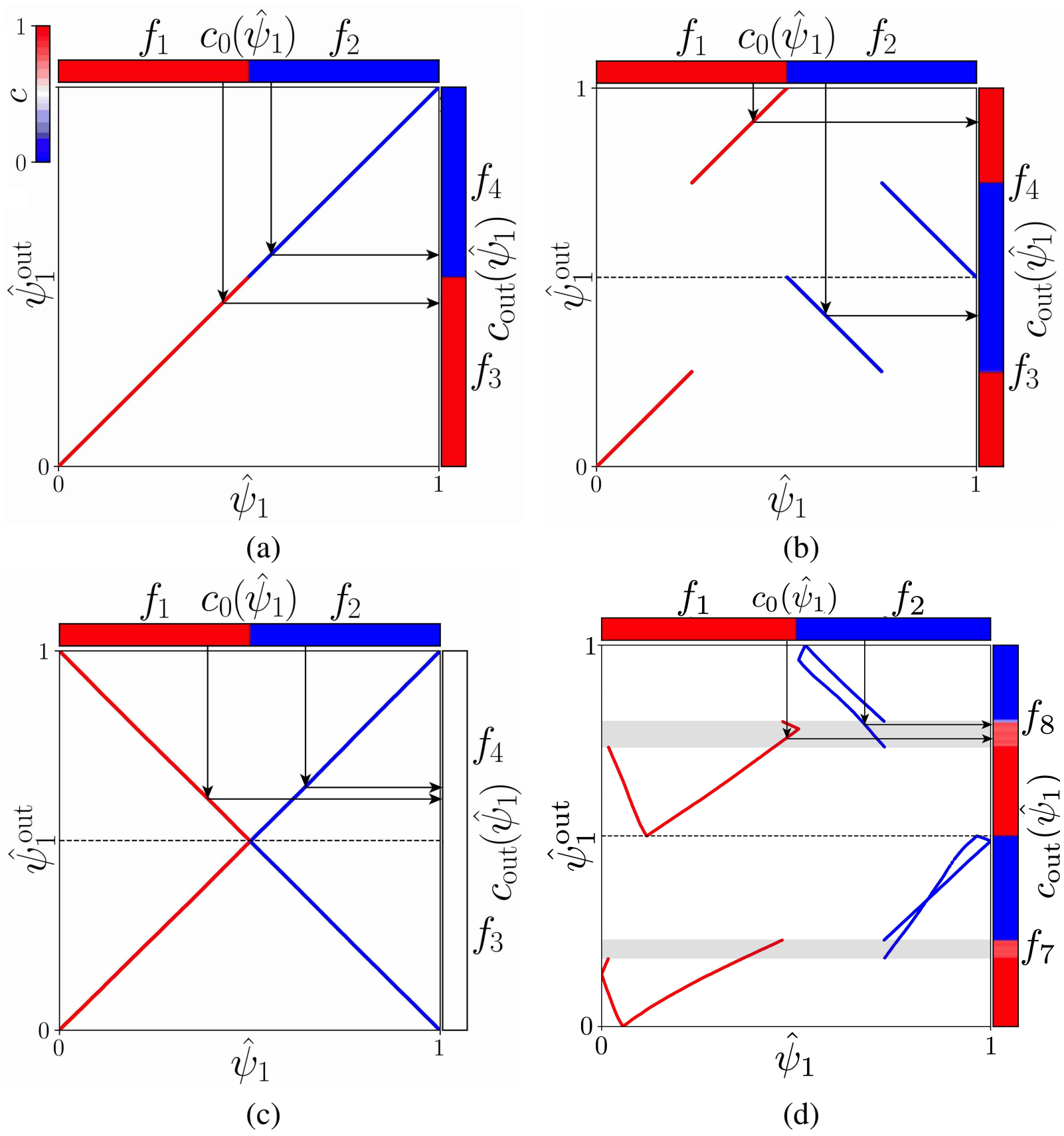

Figure 18: Examples of 1D mixing plots for fracture networks that exhibit (a) no mixing ($\hat{\psi}_1^{\text{out}} = \hat{\psi}_1$), (b) fracture mixing only (Figure 2(a)), and (c) intersection mixing only (Figure 2(c)). The top horizontal strip on each plot shows the inlet 1D concentration distribution $c_0(\hat{\psi}_1)$ and the right vertical strip shows the outlet 1D concentration distribution $c_{\text{out}}(\hat{\psi}_1)$ that correspond to the 2D concentration fields shown in Figure 2(b,d) and the associated faces are denoted f_n. Each plot shows the global streamfunction mapping $\hat{\psi}_1 \mapsto \hat{\psi}_1^{\text{out}}$ (indicated by black arrows) that also maps $c_0 \mapsto c_{\text{out}}$, and each point (which is coloured with respect to its inlet concentration value c_0) in the $\hat{\psi}_1 - \hat{\psi}_1^{\text{out}}$ plane corresponds to a given streamline, hence continuous sets of streamlines form lines in the 1D mixing plot. Fracture mixing in (b) acts to macroscopically cut and shuffle continuous segments of lines along $\hat{\psi}_1^{\text{out}}$, whereas intersection mixing in (c) acts to microscopically cut and shuffles individual streamlines with respect to $\hat{\psi}_2^{\text{out}}$ (not resolved), leading to "overlap" (grey region) of streamlines with the same ψ_1^{out} but different c_0 values that thus generate $1 < c_{\text{out}}(\hat{\psi}_1) < 0$. (d) 1D mixing plot for the fracture network shown in Figure 1b and its corresponding mixing graph $\mathcal{G}_M$ representation in Figure 10. Horizontal and vertical bars respectively indicate inlet $c_0(\hat{\psi}_1)$ and outlet $c_{\text{out}}(\hat{\psi}_1)$ concentration distributions, f_n indicates inlet and outlet faces, coloured lines in the $\hat{\psi}_1 - \hat{\psi}_1^{\text{out}}$ plane indicate streamlines that are cut and shuffled via both fracture and intersection mixing, and gray bars indicate "overlap" regions where intersection mixing occurs (and hence dilution of $c_{\text{out}}(\hat{\psi}_1)$).

manifests as half the points along this line being reflected along the $\hat{\psi}_1^{\text{out}} = 1/2$ line, leading to multiple streamlines (points) with the same $\hat{\psi}_1^{\text{out}}$ streamfunction but different $\hat{\psi}_1$ (and thus c_0) values. This "overlap" with respect to $\hat{\psi}_1^{\text{out}}$, which does not occur under fracture mixing (Figure 18b), is a clear signature of intersection mixing. Here the $c = 0$ and $c = 1$ streamlines have the same $\hat{\psi}_1^{\text{out}}$ value,

and so these concentrations are averaged according to (3.22), leading to dilution of the outlet 1D concentration $c(\hat{\psi}_1^{\text{out}}) = 1/2$ distribution, which is homogeneous according to the mixing measures $\phi^2_{\text{1D}}(s) = \Phi^2_{\text{1D}} = 0$. Note that the fully resolved outlet 2D concentration distribution (Figure 10d) has concentration values limited to $c = \{0, 1\}$, and so its variance is unchanged. This discrepancy is solely due to the microscopic nature of intersection mixing - as it mixes at length scales $s \lesssim \ell$, it is *represented* as fully mixed under the macroscopic 1D representation $s > \ell$ but unmixed under the 2D representation. As per (3.23), under such intersection mixing, $\phi^2_{\text{1D}}(s) = 0$ and $\phi^2_{\text{2D}}(s) \neq 0$ for $s < \ell$, but $\phi^2_{\text{1D}} = \phi^2_{\text{1D}} = 0$ for $\ell < s < L$, hence $\Phi^2_{\text{1D}} \approx \Phi^2_{\text{2D}}$.

Figure 18d shows the 1D mixing plot for the fracture network shown in Figure 1b and the corresponding 2D concentration fields shown in Figure 10. The outlet faces in Figure 10 show clear evidence of both fracture mixing (CS with respect to $\psi_{1,n}$) and intersection mixing (CS with respect to $\psi_{2,n}$). Similarly, the 1D outlet concentration field $c_{\text{out}}(\hat{\psi}_1)$ in Figure 18d shows clear evidence of CS along $\hat{\psi}_1^{\text{out}}$, and dilution due to CS along $\hat{\psi}_2^{\text{out}}$. Similarly, the mapping of lines in the $(\hat{\psi}_1, \hat{\psi}_1^{\text{out}})$ plane show signatures of both fracture mixing from Figure 18b and intersection mixing from Figure 18c, the latter of which leads to the overlap regions shown in Figure 18d and dilution of $c_{\text{out}}(\hat{\psi}_1)$.

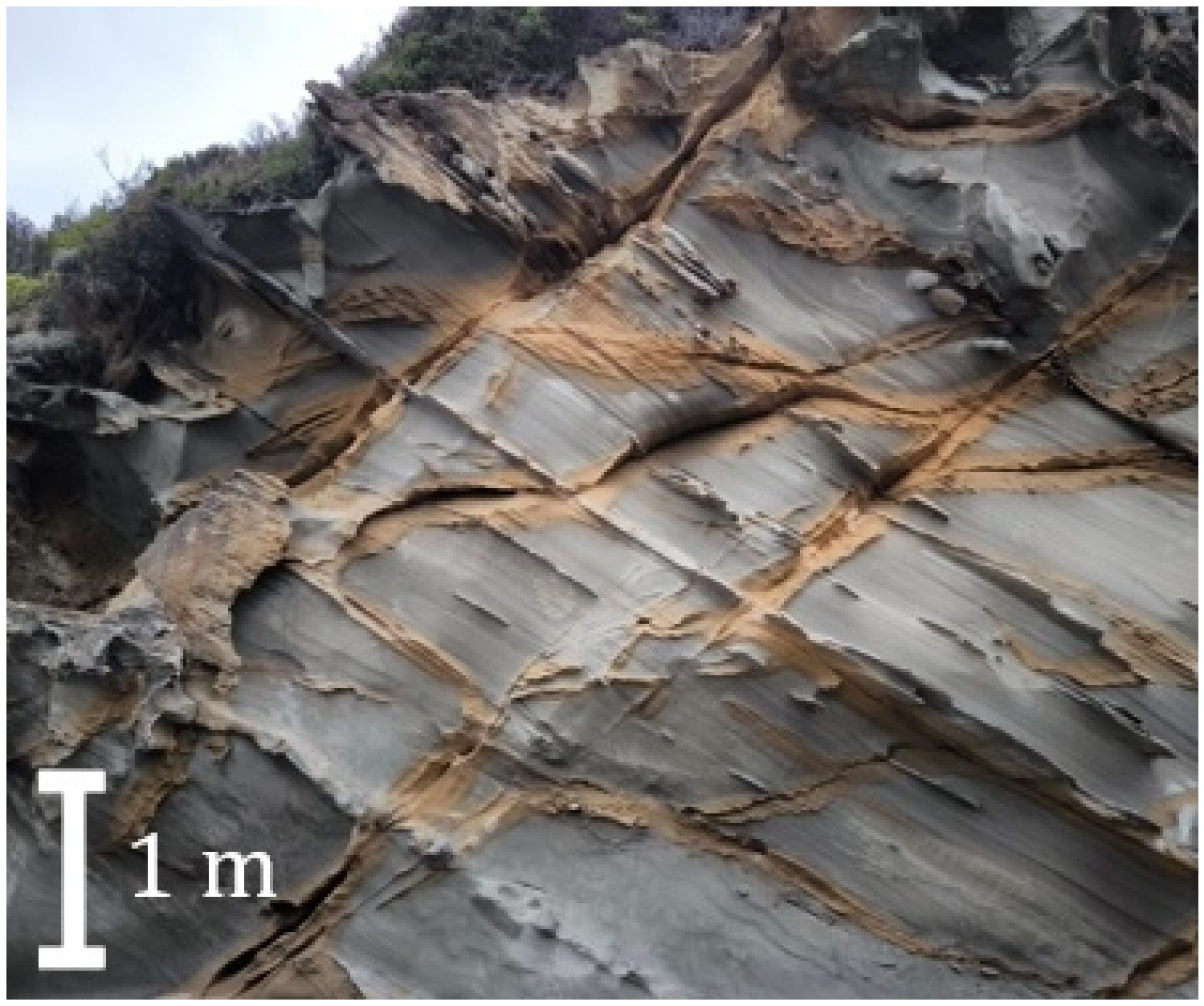

Fractured rock in Cape Otway, Victoria, Australia. Orange bands depict regions of chemical activity.

130x109mm (72 x 72 DPI)

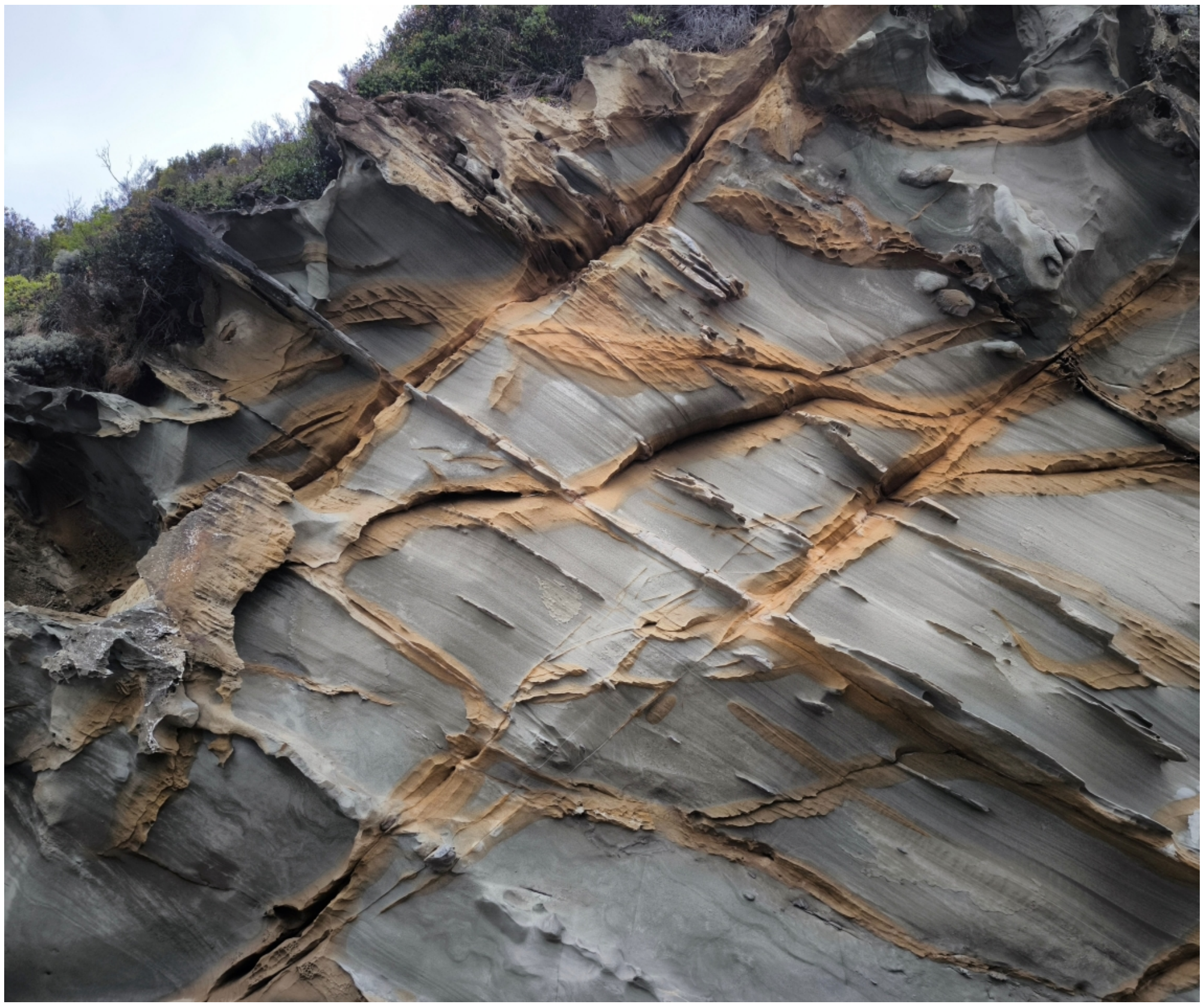

1294x1078mm (72 x 72 DPI)
